# The Development and Acceptability of a Wilderness Programme to Support the Health and Well-Being of Adolescent and Young Adult Cancer Survivors: The WAYA Programme

**DOI:** 10.3390/ijerph191912012

**Published:** 2022-09-22

**Authors:** Miek C. Jong, Trine Stub, Eric Mulder, Mats Jong

**Affiliations:** 1 National Research Centre in Complementary and Alternative Medicine (NAFKAM), Department of Community Medicine, Faculty of Health Sciences, The Arctic University of Norway, UiT, Hansine Hansens veg 18, 9019 Tromsø, Norway; 2 Department of Health Sciences, Mid Sweden University, Holmgatan 10, 851 70 Sundsvall, Sweden

**Keywords:** ecosophy, expedition, health promotion, mindfulness, nature, self-realization, qualitative study

## Abstract

Detailed descriptions of theory, structure, and activities with causal links to specified outcomes of wilderness programs are lacking. Addressing this gap, the present qualitative study gives a thorough description of the development of the Wilderness programme for Adolescent and Young Adult (AYA) cancer survivors (WAYA). WAYA is adapted to the individual needs of AYA cancer survivors. It was conceived around Næss’s ecosophy and the Positive Health Model, and refined based on findings from a scoping review and patient/public involvement. Programme aims were to increase physical activity, self-confidence, personal growth, joy, safety within nature, meaningful relationships, and self-efficacy. The programme was an eight-day expedition followed three months later by a four-day base-camp. Activities included hiking, backpacking, kayaking, rock climbing, mindfulness and bushcrafting. Evaluation of the programme through focus group and individual interviews with 15 facilitators and 17 participants demonstrated that a diverse group of participants, challenging activities, and mindfulness-based practices were found to positively influence group bonding and the learning process. Furthermore, including an expedition and base-camp component was found to be beneficial in supporting the development of participants’ own personal outdoor practices. In conclusion, this study demonstrated that the WAYA programme is safe and well accepted by AYA cancer survivors.

## 1. Introduction

*“The smaller we come to feel ourselves compared to the mountain, the nearer we come to participating in its greatness”.*

Arne Næss

Given our long tradition of living as hunter-gatherers, we are in principle adapted and equipped to live in close contact with nature [1]. However, urbanization and our technology-driven lifestyle has resulted in increased disconnection from nature [2,3]. In the last decade public health authorities have started recognizing the role nature plays in support of both personal health and population-based upstream health promotion strategies [4]. Contact with nature is associated with better health and well-being, with links to improved cognitive function, brain activity, blood pressure, mental health outcomes, physical activity, and sleep quality [5,6]. Health benefits are seen as a result of spending as little as two hours per week in a natural environment [7]. Several potential mechanisms may be involved in observed health improvements that result from time spent in nature [8]. Reports indicate that a green environment mitigates the negative impact of noise, air, and heat pollution [9,10]. Exposure to nature may also support increased physical activity [9] and enhance the associated health benefits [11]. In addition, natural environments are shown to facilitate social contact and enhance feelings of community [12,13]. According to Kaplan’s Attention Restoration Theory [14], time in nature decreases the effort required for brain function, thereby promoting recovery from mental fatigue and subsequent attention restoration. Magnetic resonance imaging studies show that time spent outdoors is positively associated with grey matter volume in the right dorsolateral prefrontal cortex and also with positive affect, independent of possible other nature-related benefits such as physical activity [15]. Additionally, time in nature is associated with changes in gut microbiota and subsequent immunological benefits [16,17]. Taken together, the range of potential mechanisms which underlie the health promotion effects of nature point to a complex process that requires further investigations in specific target populations and nature settings.

One population that benefits from contact with nature is cancer patients. A systematic review and meta-synthesis showed that time in nature supports patients in navigating the clinical and personal consequences of cancer [18]. A particularly vulnerable subpopulation of cancer survivors are adolescent and young adults (AYA), defined by the National Cancer Institute as between 15 and 39 years of age [19]. The majority of AYA cancer survivors experience long-term and late effects from cancer or cancer-related treatment [20], such as concentration/memory problems, fatigue, secondary malignancies, cardiovascular disease, endocrine and sexual dysfunction, fertility issues, body disfigurement and mental health issues [21]. As AYA cancer survivors are at a stage in life where they are navigating studies or early career, maintaining friendships, building meaningful relationships, and managing finances, these long-term and late effects pose particular physical, emotional, cognitive and social challenges to this population [21].

Adventure therapy programmes have been designed for AYA cancer survivors to meet their unique psychological and social needs. They offer physically challenging adventure and group activities for specified therapeutic outcomes [22]. Adventure therapy for cancer survivors originates from specialized summer camps for children and youth with cancer, which often take place in an outdoor or nature setting [23]. A recent scoping review on wilderness programmes for childhood cancer survivors, including outdoor-based adventure therapies, described positive effects of these programmes on social involvement, self-esteem, self-confidence, self-efficacy, social support, and physical activity [24]. Although nature had a contextual and therapeutic premise in all wilderness programmes included in this scoping review, it became apparent that the role of nature and underlying theoretical concepts were given little attention [24]. Similar findings were recently reported by Harper et al. [25] in an umbrella review that explored the role of nature in outdoor therapies. They found that clear and comprehensive descriptions of theory, programme structure and activity details with causal links to specified outcomes are mostly absent in scientific literature on outdoor therapies. It was concluded that a rigorous and focussed programme of research is required to explicitly address in-depth theories of change, the type and characteristics of nature included, and the manner in which natural environments are used to support the change [25]. Other gaps in the field of outdoor research include absence of randomized and long-term studies, and thorough investigation of safety considerations for young cancer survivors [24,25]. Additionally, most outdoor programmes were developed in the USA, and none were found to be available to AYA cancer survivors in the Scandinavian countries [24]. Therefore, the present study was initiated with the following aims:

1. To systematically develop a Wilderness programme for AYA cancer survivors (WAYA programme) in the Scandinavian countries, including theory, concept, and content building.
2. To investigate the acceptability of the wilderness programme among facilitators and AYA cancer survivors.

Related to the first aim of this study, the ecosophy of Arne Næss [26] was used as a theoretical framework to develop the WAYA programme. Central to Næss’s ecosophy is the idea that every living being has an inherent drive to expand its power and fulfil its potential—which he refers to as self-realization [27]. In this context, Næss does not intend “self-realization” to be of the ego self (small s), but rather a realization of the larger ecological Self (capital S). Identification of the individual with other living and non-living beings with whom we are bidirectionally connected is one path to realization of the ecological Self. According to his view, all beings are connected in the dynamic web of nature, where each has its own intrinsic value that should be recognized and respected [1]. Time spent in a natural setting can support the process of realization of the ecological Self [27]. The programme provides participants the opportunity to identify with and establish a connection to the beauty and grandeur of nature, thereby supporting the expansion of a larger and more inclusive sense of ecological Self. According to the initiators of the WAYA programme (first and last author) this shift is hypothesized to be supportive of an increased sense of their well-being. Inherent in the shift to this new orientation is a sense of personal growth and meaning-making, both of which have been linked to eudaimonic well-being. This in turn is thought to be of great importance in the ability to deal with significant life challenges such as surviving cancer [28].

To investigate the acceptability of the wilderness programme among facilitators and AYA cancer survivors, a qualitative study design was chosen and data collection was made with (focus) group and individual interviews.

This study will inform young cancer survivors, outdoor instructors, nature organizations, public health actors, and researchers about the theory, health concept, content and health promotion aims of a wilderness programme for AYA cancer survivors, and provides recommendations on how to optimize future programme planning and research.

## 2. Material and Methods

### 2.1. Study Subjects

Study subjects were facilitators and AYA cancer survivors.

#### 2.1.1. Facilitators

A total of 15 facilitators were recruited for the WAYA programme via the outdoor network of the first and last author. Prior to the start of the programme, all facilitators were briefed about their designated tasks and responsibilities, the study protocol, the risk and safety plan and how to keep a field diary for research purposes. Two medical doctors acted as remote advisors, with 24-h availability to consult regarding any medical issue if needed. The demographic characteristics and competences of facilitators are shown in Table 1.

#### 2.1.2. Participants

The WAYA programme was targeted towards AYA cancer survivors aged 16 to 39 years. There were no limitations for type of cancer diagnosis, nor for when they received their diagnosis or treatment. The content of the WAYA programme required participants to have the ability to walk two kilometers without pausing. Experience with outdoor life was not necessary, and the programme was individually adapted according to the individual needs and interests of the participants. Participants were recruited via the national cancer associations, Ung Cancer and the Swedish Childhood Cancer Fund [29]. The demographic characteristics of the 17 participants that were interviewed for evaluation of the WAYA programme are shown in Table 2.

Participants were between 20–35 years of age (median 30), mostly female (71%) and had several varying cancer diagnoses, age at cancer diagnosis, and time since treatment (Table 2). All 17 (100%) participants reported health issues relating to long-term and late effects of cancer (treatment), although it cannot be excluded that some health issues had a different origin. The most prevalently reported health issue was pain (*n* = 12, 70.6/%) followed by mental health problems (*n* = 10, 58.8%), allergies/asthma (*n* = 6, 35.3%), cognitive dysfunction (*n* = 5, 35.5%), depression (*n* = 5, 29.4%), gastrointestinal problems (*n* = 4, 23.5%) and anxiety (23.5%) (Table 3).

### 2.2. Programme Development

The development of the WAYA programme was the first step in the planning for a pilot randomized controlled trial (RCT) on the acceptability, feasibility, safety, and impact of such a programme among AYA cancer survivors. The protocol for this mixed-methods pilot RCT study has been registered in a clinical trial register (NCT04761042) and has recently been published [29]. Results of the RCT will be published elsewhere at a later stage.

**Figure 1:**
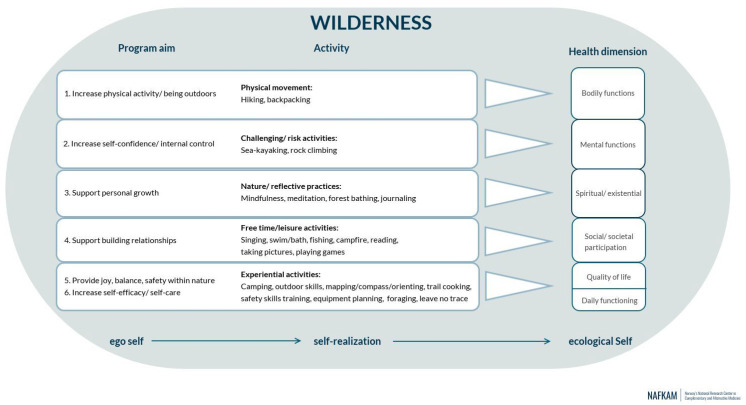
Schematic overview of the WAYA programme.

The development of the WAYA programme included a step-by-step systematic approach involving: (1) The theoretical foundation; (2) Concept building and formulation of aims by means of the Positive Health Model; and (3) Content building through a scoping review and patient and public involvement. The WAYA programme is schematically presented in Figure 1.

#### 2.2.1. Theoretical Foundation, Concept Building and Aims of the Programme

The theoretical foundation and underlying health promotion principle of the WAYA programme is support for the self-realization process of AYA cancer survivors according to the ecosophy of Arne Næss [26] (see Figure 1). The Positive Health Model as developed by Huber et al. [30,31] was used as a conceptual framework to further guide the health promotion design of the WAYA programme. The Positive Health Model is an operationalization of the dynamic health concept that describes health as “the ability to adapt and to self-manage, in the face of social, physical and emotional challenges” [32]. In practice, this model reflects a holistic view of health that includes six health dimensions: bodily functions, mental functions and perception, spiritual/existential dimension, social and societal participation, quality of life, and daily functioning [30]. The conceptual relationship between “health as the ability to adapt and self-manage” and the six “health dimensions” has been explained in the way that the experienced quality of the latter can inform to what extent a person is healthy, i.e., is able to adapt and self-manage [33]. The aims of the WAYA programme were formulated according to each of the six health dimensions to support the health and well-being of AYA cancer survivors from a broad perspective (see Figure 1).

The six aims of the WAYA programme are:

1. To increase physical activity in the context of the natural environment
2. To increase self-confidence and provide an increased perception of internal control
3. To support personal growth
4. To foster togetherness, a sense of community, and support the building of meaningful relationships
5. To provide joy, balance, and safety within the grandness of nature
6. To increase self-efficacy and self-care in the context of personal health and well-being.

#### 2.2.2. Content Building

A scoping review on wilderness-related programmes for childhood cancer survivors was performed to assist in the development of the content of the WAYA programme [24]. From the 15 studies that were included in the review, a total of five programme content-related categories were synthesized: (1) challenge/risk activities; (2) free time/leisure activities; (3) experiential learning activities; (4) physical activity; and (5) psychotherapeutic work. For the WAYA programme activities from the first four categories were selected, while activities related to psychotherapeutic work were excluded. The reasoning for this was that the WAYA programme was initiated and developed from a health promotion perspective and not as a cancer-related (psycho)therapy. In addition another category of programme activities was added based on the recommendations in this scoping review [24], that was explicitly directed towards reflective practices in the natural environment [29]. This category included the following formal (guided) and informal exercises: daily check-in and check-outs for (group) reflection, forest bathing [34], journaling, mindfulness, meditation (walking meditation, silent kayaking, 5-senses meditation, loving-kindness meditation [35]). The content of the WAYA programme was further developed through patient and public involvement by way of input and feedback on programme activities from representatives/members from the national cancer associations (Ung Cancer (*n* = 3) and The Swedish Childhood Cancer Fund (*n* = 2)), as well as from wilderness/outdoor instructors (*n* = 2) from the Swedish Survival Guild. An overview of the different programme activities in the WAYA programme is schematically presented in Figure 1, with additional detail in Supplementary Materials S1 (Tables) Field diary.

#### 2.2.3. The Role of Nature in the WAYA Programme

Due to an apparent literature gap in relation to the role of nature in wilderness programmes [24,25], considerable attention was paid to the topic in the development of the WAYA programme. To start, the programme was grounded in the ecosophy of Næss [1], whereby nature plays a contextual role in support of the development of a sort of relationship with other beings in nature. A diversity of landscapes was included in the WAYA programme in order to create an opportunity for participants to experience the uniqueness of different biotopes as well as to allow them to explore relationship and affinity to each one. These included boreal forest, wetlands with marsh vegetation, rocky heights and plateaus, sandy beaches, islands, lakes, and open sea. Secondly, specific characteristics of the wilderness settings were utilized for activities in support of the health promotion aims of the WAYA programme. Examples of such activities include hiking, rock climbing, and sea kayaking, which were chosen to pose certain challenges for participants (related to aim numbers 1, 2, 5 and 6, see above). Hiking included such challenges as crossing streams and navigating hazards like tree roots, fallen trees, branches, loose stones, and slippery rocks and mud. Climbing a gorge entailed use of ropes, harness, carabiners, and specific climbing knots. Kayaking posed challenges such as balancing the boat, working in harmony with another paddler in the double kayak, exposure to water temperatures as low as 5 degrees Celsius, and navigating potential strong wind and currents. Exposure to old-growth forests within the national park allowed comparison with recent clear-cuts around the borders of the park, so that participants could experience the impact of humans on nature. Aspects of the wilderness setting were also used to support the mindfulness exercises, in line with programme aim numbers 3–5. These included forest bathing [34] along a valley of an old-growth forest, silent walks, attentive listening, and silent kayaking along a coastal forest, all of which were intended to encourage awareness practice, support an experience of balance and harmony within the natural surroundings, and allow an opportunity to share these experiences and their thoughts about them with other members of the group during daily check-in and check-outs. Programme aim number 6 was primarily addressed by way of teaching bushcraft skills. Lakes and streams were used as sources of drinking water, lakes and forests provided food (fish, mushrooms, berries), forests provided wood for cooking and campfires, and sandy beaches were used to construct a steam pit for outdoor cooking. Finally, in support of WAYA programme aim numbers 3 and 5, kayaking to and camping on an uninhabited island was included to allow the experience of feelings of solitude and distance from civilization. Hiking to the peak of the highest island in Sweden toward the end of the wilderness expedition provided the opportunity for a metaphorical visualization of all aspects of the journey the participants had undertaken together before summiting and returning home.

#### 2.2.4. Structure of the Programme

The WAYA programme was composed of an eight-day wilderness expedition experience, followed by a four-day base camp experience three months later (see Table 4). The learning goal of the wilderness expedition was the development of backpacking skills, including carrying all necessary gear, camp set up and breakdown, and learning “leave no trace”. A specific learning goal of the base-camp part of the programme was development of more in-depth bushcraft skills, including different ways to make a fire and additional food collection and preparation skills. In the three-month period between the expedition and base-camp, participants were coached in the practice of their own outdoor activities in two online sessions. Throughout the WAYA programme all necessary outdoor equipment, outdoor clothing, footwear, and meals were provided to participants without cost. All outdoor equipment could be borrowed and taken home by participants for further use in establishing their own outdoor practice. During the expedition, meals consisted of freeze-dried food pouches to which only (hot) water was added before consumption. The content/taste of the freeze-dried meals varied considerably and included meat, fish, vegan, and lactose- and gluten-free options. The average number of calories per freeze-dried meal was 431 kcal for breakfast and 559 kcal each for lunch and dinner. Additionally, participants were provided daily with fresh fruits, dried fruits, nuts, protein bars (160 kcal), coffee, tea, and juices. Toward the end of the expedition, a taco dinner was prepared by all participants which included fresh vegetables. During base-camp, freeze-dried food pouches were alternated with freshly prepared steam pit meals and meals cooked over a wood-fire or in a wood field stove. These meals included ingredients that were harvested by participants from nature that day.

As shown in Table 4, the maximum group size for the expedition and base-camp was ten participants. The group structure for both programme parts was closed, meaning that no new (other) participants entered the programme after the start. The amount of time per day participants spent in group or structured activities per day, daily free time, and facilitator/participant ratios are listed in Table 4. Free time was defined as the time where participants were not involved in any programme-offered group or individual activity.

Group and structured activities in the WAYA programme were individually adapted according to the participants’ physical and/or mental capabilities, and were also adapted to their personal interests (see Table 5).

#### 2.2.5. Setting

The WAYA programme took place in the wilderness setting of the High Coast in Sweden. This area is considered to be little influenced, manipulated or impacted by humans, in line with the description of wilderness by Næss [26]. The expedition programme was first piloted among facilitators in April 2021, in the area south-west /south of Skuleskogen National Park and on the island of Mjältön. The planned hiking and kayaking routes were tested, carefully inspected, and discussed among facilitators in relation to the capabilities of AYA cancer survivors and different weather conditions. Adjustments were then made to planned camping sites, kayaking routes, and alternative routes. The two separate expeditions with two different groups of AYA cancer survivors (*n* = 10 and *n* = 9 participants, respectively) took place in June 2021. The two separate base-camps with the same groups of participants (*n* = 8 and *n* = 9 participants, respectively) took place in September 2021. It should be noted that the Swedish “Allemansrätt” (freedom to roam) allows people to camp freely in nature. However, using nature/wilderness with an overnight stay for commercial purposes or for group activities is not allowed. Hence, all involved landowners where camp was set up were contacted prior to the start of the study and permission to use their nature property was obtained.

### 2.3. Risk and Safety Plan

A risk and safety plan for the WAYA programme was developed according to the risk exposure/consequence matrix of the Swedish Mountain Society [36], and input of patients and the public [29]. The objective of the plan was to identify potential risks or safety issues related to the programme and then take the corresponding precautions and make preparations to prevent potential injuries or accidents. Since the WAYA programme was piloted between the third and fourth wave of the COVID-19 pandemic (June–September 2021), specific attention was paid to COVID-19 testing and hygiene measurements. Upon arrival and then again on the second day of both the expedition and base-camp programmes all participants and facilitators were tested for COVID-19 infection. During the programme further COVID-19 testing was performed if a participant or facilitator exhibited COVID-19 related symptoms. All participants were provided with face masks and disinfection wipes during transportation to the wilderness site when social distancing was not possible. According to original programme planning, each tent was meant to be shared by two participants. However, due to the COVID-19 pandemic all participants were provided with their own tent. The risk and safety plan of the WAYA programme is attached in Supplementary Materials S2 (Document). For ethical reasons, the names of facilitators and telephone numbers have been omitted from the document.

### 2.4. Programme Evaluation

The evaluation of the WAYA programme had an exploratory qualitative approach and aimed to obtain a deeper understanding of the topic under study (here; in terms of acceptability of the wilderness programme among facilitators and AYA cancer survivors). The empirical data was collected through semi-structured field diaries (facilitators), a focus group interview (facilitators) and through individual interviews with participants. All interviews were transcribed verbatim and subsequently analyzed using a qualitative content analysis [37].

#### 2.4.1. By Facilitators

The acceptability of the WAYA programme was evaluated by facilitators by means of a field diary (Supplementary Materials S1 (Tables)) and a focus group interview. A field diary was developed and provided to each facilitator for the purpose of evaluating the programme. Facilitators were contacted by email and invited to participate in the focus group interview. A total of ten out of 15 facilitators responded and gave informed consent for their participation. The focus group followed an interview guide (see Supplementary Materials S3 (Document)) that was developed based on domains that were identified through an inductive content analysis of the field diaries [37]. The focus group interview took place online in November 2021, two months after the base-camp programme, and lasted for 1.5 h. The interview was conducted in English, recorded, and transcribed verbatim for further analysis.

#### 2.4.2. By Participants

Participant evaluation of the acceptability of the WAYA programme was done by means of interviews. An interview guide was developed in which participants were asked to describe their overall impression of the wilderness programme, and also their more specific perceptions of the different programme components, activities, and facilitators (see Supplementary Materials S4 (Document)). About 3.5 months after the first expedition (two weeks after base-camp), an invitation to be interviewed was send to 19 participants via email. Seventeen of the 19 participants responded, and subsequently 17 online interviews were conducted by the second author (who was not otherwise involved in the wilderness programme), within the two-weeks following the email invitation (September–October 2021). The interviews had an average duration of 44 min (range: 21–74 min) and were recorded and then transcribed verbatim. An inductive content analysis was performed on the field diaries of the facilitators, where five domains were identified. The domains are described in the introduction of the results. Subsequently, a deductive manifest analysis was carried out regarding participants and facilitators “perceptions” of the different activities of the programme relating to the identified domains [37].

### 2.5. Ethical Considerations

Participants were informed both orally and in writing that participation in the study was voluntary. In addition, it was made clear that participation in all programme activities was voluntary as well, that participants could decline participation in any activity without explanation, and that they could withdraw at any time from the programme without stating a reason. Participants were further informed about the purpose of the study, that data would be handled and later published/presented in a confidential way. Informed consent was obtained from all participants prior to the start of the study. All participants and facilitators were insured in case of unexpected programme-related accidents. Participation in the WAYA programme was without any costs for participants.

## 3. Results

Five domains with relevance for the acceptability of the WAYA programme were identified: (1) Type of wilderness programme; (2) Activities in the programme; (3) Safety of the programme; (4) Facilitators in the programme; (5) Equipment in the programme. The perceptions and experiences of both facilitators and participants are further described for each of these domains.

### 3.1. Type of Wilderness Programme

First, perceived advantages and disadvantages of the expedition versus a base-camp programme are described. The facilitators’ perceptions were that participants were challenged by the expedition activities and that the challenges were big enough to allow participants to feel a sense of accomplishment in realizing they were able to achieve certain tasks. Participants expressed that the expedition created opportunities for the group to bond and to support one another in overcoming the challenges.

*“…first and foremost, it was that we were a group hiking together, it’s a lot easier when you are a team… one day we hiked about ten kilometres or similar, it was far and took a whole day but since we were a team supporting and encouraging each other and having fun, we could really have hiked 40 kilometres.”*

(Participant 10)

Some facilitators perceived that there was too little free time and perhaps little motivation for participants to socialize during the expedition, but this was not confirmed through the interviews with participants. Only one participant expressed a need for more free time during the expedition, but also acknowledged that this probably would require another form of programme:

*“I understand that I am not on a spiritual camp, but on a camp to hike along with other people touched by cancer. So in case I would want more relaxation, it would have been more appropriate with a retreat for ten days or so…”*

(Participant 18)

Furthermore, facilitators considered that the pace was high during the start of the first expedition programme, which did not suit all participants. The hiking distances for the first two days of the second expedition were therefore shortened by two, respectively three kilometers to allow for more free-time. The base-camp, on the other hand, was included in the WAYA programme to allow more time for relaxation, for social interaction with others in the group, and to allow time for learning specific bushcraft skills, but was less varied with respect to landscapes and challenging activities. Overall, facilitators thought it was an advantage to have both types of programme settings included (see example below).

*“I believe that one of the advantages to the expedition is kind of getting through the challenges of it together, so that participants have an opportunity to help one another and, build their relationships…. therefore, when they get to base-camp follow up, they can….—I love the idea that there was both actually -…. kind of solidify the relationships started with the expedition …. and have the space to talk and hang out.”*

(Facilitator 8)

### 3.2. Activities in the Programme

Given the large variation in the physical capacity and functioning of participants, it was necessary to individually adapt the planned group activities in the programme. Facilitators agreed that for the group process it is important to have all participants together in the beginning of the expedition, so that everyone feels included. Later in the expedition it was seen to be of importance to have individual adaptation, since those who were more fit also wanted to challenge themselves and try new things. A cross-cutting theme that appeared from all interviews with participants was “the possibility of choice and individual adaptation”, where at times they could make choices to opt out or to do alternative activities depending on individual variation, how they felt on the day, and functional disabilities, as exemplified below.

*“Since I don’t have any distance vision it affects me in a way that I need a guide, and I need to fully concentrate. On our first hike we hiked on a narrow inclining path with lots of tree roots and stones, then I needed double concentration which was exhausting. At the same time, the rest of the group moved ahead, and I was left behind with the guide which was a bit sad, since I felt outside the group… and the second hike was too long, it was ten kilometres, so I could not make it, it would take too long.”*

(Participant 11)

In a similar vein, participants indicated enjoyment with regard to the hikes, but varied in terms of specifics. For example, some expressed a wish to hike much longer distances, and some wished for shorter distances, or even opted at times to go by car or boat between the trailheads. Facilitators also observed that those participants with neuropathic pain, balance problems, or impaired visibility were perfectly able to travel longer distances through wilderness areas by kayak. An important learning aspect of the expedition planning from the perspectives of facilitators was to have at least two alternative options for daily routes or activities. Then, it was up to participants to decide which alternative they were up for, rather than facilitators deciding for them. Unfortunately, the WAYA programme was not successfully adapted to the physical capacity and functioning of all 19 participants. Of the ten participants in the first wilderness expedition, two had to leave the expedition after three and four days, respectively, due to persistent back/neck pain and gastro-intestinal pain from irritable bowel syndrome. These pain complaints were already present before the start of the expedition, and individual adaptation of the programme could not ease their pain and related anxiety. If the pace in the beginning of this first expedition had been slower and weather conditions more favourable, these two participants may have been able to continue. The weather conditions during the second expedition were much better than during the first expedition, and with a slower pace at the start, all nine participants in the second group succesfully completed the expedition. One of the two participants who left in the first group returned for the follow-up base-camp programme; the other did not because of an unexpected family event prior to the start of base-camp. In addition, another participant in the first group was unable to travel and participate in the follow-up base-camp programme due to COVID-19 symptoms.

A large diversity of participants was considered an advantage by facilitators, even though it required more adaptation of the expedition and increased logistic support. The quote below exemplifies how the challenges contributed to learning:

*“I also think that it is one of the beauties that people have so different needs and learn from each other and learn to take care of each other, so I think it was a good mix, because if you only had chosen the most fit participants, we would have lost a good teaching opportunity for the whole group.”*

(Facilitator 4)

Another aspect that was addressed is the balance between programme-related activities and personal free time. Analysis from the data in the field diaries of facilitators showed that the free time per day during the expedition was 3.2 h on average in both groups. Facilitators expressed that the way time is spent is also inherent to the type of programme (expedition versus base-camp), since there will obviously be less free time on an expedition due to the activities involved.

*“but if you have an expedition, and you have a goal to reach the top of, whatever you want to reach, in my experience there’s not much free time. Free time is when you are there, and if you are there earlier, well you have more free time, and then your free time is cooking and chatting, and maybe checking your environment and then going to bed.”*

(Facilitator 1)

The balance between time spent in facilitator-supervised activities versus time spent on activities on their own was also considered. At the beginning of the expedition, facilitators felt that they knew little about participants or their abilities and did not know whether they would be able to manage by themselves. Supervision of participants required a delicate balancing act, where the facilitator had to consider safety together with the participants’ freedom of choice, as described below:

*“she came up to me and said: “I just want to take a stroll, going towards the waterfall”, and I was thinking…. what I have always been taught… like OK, is that safe? Is it safe for her to wander off? I do not know her.”*

(Facilitator 10)

The participant mentioned above also reflected on this balance issue, saying she felt safe and also did not feel as though she was being monitored:

*“there was a time when I went off for meditation on my own, I let them know before I left as I knew I should do so …., but then I stayed a bit longer than I had said and one of the facilitators checked on me to see whether I was ok… I understand it was out of concern for me, and I know that I am in general terrible at keeping time, and then especially while being in nature without a clock!”*

(Participant 16)

According to facilitators, clear explanations and instructions are needed so that participants gain an understanding of the importance of staying together as a group while doing outdoor activities such as hiking or paddling, with one person up front and one in the back regularly checking to be sure the group is complete. Later in the expedition there can be the possibility for participants to take hikes on their own initiative, but they must always let facilitators know where they are heading. According to the facilitators, there were also participants in the groups who preferred to spend their time in the company of others and did not want time alone.

The overall variance and balance of programme activities in the WAYA programme was perceived positively. None of the facilitators or participants expressed a wish for the inclusion of other outdoor activities. Additionally, all 17 interviewed participants would recommend the WAYA programme to other AYA cancer survivors.

Most participants had little prior experience with camping in nature, and the few who did also felt that being a part of the programme still provided learning opportunities. As an example, one of the participants reflected on what was learned:

*“(camping) went really well but was something out of the ordinary. Still you learned what was a good place to be and what was a less good place to be. I mean in what way the ground was. A couple of times I started out high up in tent when going to sleep and woke up in the foot end. During the night I slid down (smiling).”*

(Participant 5)

The reflective practices such as the mindfulness sessions were perceived by participants to be supportive of the development of group cohesion, relaxation, and connection with nature. One of the participants described how daily mindfulness sessions supported the process of group cohesion:

*“We became a group very fast. People opened up and talked about, well it was also very emotional, and at the same time when you come there and meet people who have been ill and talk about their illness there are lots of emotions, and you are allowed to just be yourself instead of trying to be someone else. That was nice… and emotional.”*

(Participant 10)

Others also perceived it as difficult when participants chose to share and talk to a deeper extent about the difficulties they had been going through:

*“it was hard for me to sit in the ring and share after the mindfulness exercises, even though what is shared is true, it is hard. People got deeper and deeper and were more and more affected. Finally, I said that if this is how it is going to be, I am not going to join in on the sharing unless it becomes sharing of something that is more positive.”*

(Participant 12)

One aspect of discussion among facilitators regarding the mindfulness exercises in the WAYA programme was whether they should share their own (private) challenges with the group or not. On the first wilderness expedition, two facilitators had concerns about sharing and thought that it was not appropriate to expose vulnerable participants to their own emotions. The other facilitators had the opinion that it was a good thing in being an “example”, as it facilitated the group process and connections within the group. On the second expedition it was decided that only three facilitators (instead of all ten) would take part in the daily check-in and checkout and group-based reflective (mindfulness) practices.

### 3.3. Safety of the Programme

Overall, facilitators and participants perceived the WAYA programme as safe.

One facilitator reflected on the safety aspects as follows:

*“I think, having facilitators with medical backgrounds or nurses is wonderful, and that you cannot be in the back wilderness, and be in the ocean, and be on the mountain and not have some challenges, that’s part of the… risk that they all agree to take on. So I felt, even though I tried to always write down the near misses, I felt it to be very safe.”*

(Facilitator 8)

One participant stated:

*“I had never kayaked before, and it was really frightening in the beginning. I also capsized (unintentionally) and that was scary, but directly as soon as I came up from under the water, they (facilitator 3 and 7) were there. You feel safe when someone is there to support you.”*

(Participant 10)

Two facilitators perceived that during the first expedition, participants were exposed to a higher risk of possible accidents due to the high pace, in combination with little time to eat and relax. However, no serious adverse effects occurred in the WAYA programme. A quantitative safety analysis of the WAYA programme will be reported elsewhere.

### 3.4. Facilitators in the Programme

The planned facilitator-participant ratio for the expedition was 1:2, but due to the specific needs of participants it turned out to be 1:1 in practice. Some facilitators thought the WAYA programme could benefit from having less facilitators, so that participants would be more likely to do more themselves and help each other. Other facilitators appreciated the 1:1 ratio, which allowed them to have more time with participants, rather than running off for logistical errands. The participants’ perception was that facilitators were never far away, and could provide support when necessary, in both practical matters and emotional support.

### 3.5. Equipment in the Programme

For the most part, outdoor equipment was felt to be appropriate and sufficient by both facilitators and participants. No specific items were lacking. One critical aspect that was mentioned by both facilitators and participants was the warmth of the sleeping bags.

*“there was just some really cold night, so sleeping… and I do not know if they were, but I did trade sleeping bags with several people, and they were mentioning that they were warmer, so… I think that would be the only thing, I think everyone was getting a little wet and stuff.”*

(Facilitator 8)

Although sleeping bags were selected to be warm enough down to zero degrees Celsius and night temperatures did not drop below seven degrees Celsius, several participants reported that they were cold during the night. Solutions were created by exchanging sleeping bags with facilitators, wearing more clothing during the night, insulating beneath the sleeping mat, engaging in physical exercise before going to sleep, or placing hot water bottles in the sleeping bags.

*“I learned, because I did not know that the cold comes from the ground, so I had some extra insulation under my sleeping mat, and additionally I learned that it is good to run a bit to get warm before you go to bed in the sleeping bag.”*

(Participant 16)

The freeze-dried food was generally perceived to be tasty and acceptable by participants, and dietary variation by inclusion of additional snacks, fruits and occasionally some fresh food was appreciated. However, eating the freeze-dried food for eight days in a row during the expedition appeared to give too little variation in texture and taste. Participants suggested that more opportunities for fresh food cooking in the WAYA programme would be appreciated, but also acknowledged the potential logistical challenges in providing fresh food when you are out in the wilderness.

## 4. Discussion

To the best of our knowledge, the WAYA programme is the first wilderness programme that has been developed in the Scandinavian countries for this specific target group. Næss’s ecosophy [1] provided the theoretical underpinning of the programme, which together with the six domains in the Positive Health Model [30] guided the formulation of programme aims, the use of nature in the programme, and further content building (Figure 1). The present study demonstrated that the WAYA programme is safe and accepted. An important finding of this study was the perceived added value of including a diverse group of AYA cancer survivors for group bonding and learning purposes. The AYA cancer survivors in the WAYA programme all reported having existing health-issues that may have affected their participation in the programme to some extent. It is known from the literature that most childhood and AYA cancer survivors are physically and/or mentally impacted by long-term and late effects of cancer treatment or the cancer itself [38,39]. It is therefore of crucial importance to address their various comorbidities and/or disabilities in the design stage of wilderness programme development. Yet, so far, little to nothing has been published on how wilderness programmes are adapted to meet the specific needs of AYA cancer survivors [24]. The present study describes in detail how the wilderness expedition was adapted to their physical and mental disabilities, and that this was successful for most (17 out of 19) participants. Amongst other things, it required a flexible travel schedule with sufficient possibility for choice and individual adaptation (Table 2). Furthermore, a facilitator—participant ratio of 1:1 was required to guarantee sufficient guidance and support, as well as to meet the logistical challenges inherent in alternative travel options. The majority of participants in the WAYA programme were female, which is in line with previously reported findings demonstrating that 78% of childhood cancer survivors who participate in wilderness programmes are female [24]. In wilderness programmes that target at-risk youth and those with mental health problems it is the other way around, i.e., a higher number of males than females participate [40,41,42]. In the present study, participants were mainly recruited from membership in the national cancer associations, Ung Cancer and the Swedish Childhood Cancer Fund. Both associations confirmed that it is predominantly female members who are most active in seeking support and participating in activities that are offered.

The WAYA programme is unique in the way that it included an expedition as well as a follow-up base-camp experience, both aiming to support the health and well-being of AYA cancer survivors in the long-term. This contrasts with existing programmes for this target group, which either consist of an expedition [43,44,45] or a base-camp setting [46,47,48,49]. In addition, the WAYA programme included elements from adventure programmes (such as challenging kayaking and rock-climbing activities) as well as mindfulness-based exercises, with the aim of supporting participants in development of increased self-confidence, and increased sense of internal control over their life, and further personal growth. A wilderness programme that seems to be quite similar to the WAYA programme with regard to content is True North Treks in the USA [50]. Although the True North Treks programme was much shorter (five day expedition or a three day base-camp versus an eight day expedition and four day base-camp in the WAYA programme), it also included challenging activities as well as mindfulness-based practices. A single arm within-subjects evaluation of the True North Treks programme showed improved psychosocial outcomes and changes in inflammatory biomarkers among young adult cancer survivors [50]. The impact of the WAYA programme on the mental and physical health status of AYA cancer survivors will be investigated in a pilot randomized controlled trial in which WAYA programme participants are compared to a control group of AYA cancer survivors that joined a holiday programme at a Spa hotel [29].

## 5. Strenghts and Limitations

A strength of the current study is that it describes a step-by-step and systematic approach for development of a wilderness programme targeted towards AYA cancer survivors involving patient and public participation. Where previous studies failed to specifically address the role of nature in the programme and the manner in which the natural environment was involved in support of the aims and outcomes of the outdoor programme [24,25], the present study gives a detailed description that emphasizes the importance and contextual use of nature in the WAYA programme. Another strength is that the WAYA programme was led by an international and competent group of facilitators. Furthermore, the acceptability of the programme was evaluated from both participant and facilitator perspectives. There are also some limitations of this study that need to be considered. First, the WAYA programme was piloted among two group of participants (n = 19 in total). Although the group exhibited broad diversity with respect to cancer diagnosis, time since cancer diagnosis and physical and mental status, there was an under-representation of AYA cancer survivors from other cultural backgrounds outside of Sweden. Evaluation of the WAYA programme among a more diverse group of AYA cancer survivors could thus have revealed other relevant aspects for optimization of the programme. Similarly, although the majority of participants (17/19: 89%) and facilitators (10/15: 67%) participated in the interviews and focus group, respectively, the experiences and perceptions of the non-respondents may have revealed other important insights to improve the programme. However, the overall sample size of the present study was evaluated to be sufficient to obtain a deeper understanding of the acceptability of the wilderness programme among facilitators and AYA cancer survivors. The 17 interviews with AYA cancer survivors and a focus group with 10 facilitators, plus additional field diary data, provided rich data (holding/giving relevant information) in relation to the study objective. Typically, qualitative research has between 1–30 respondents [37], and usually smaller sample sizes are accepted when data is rich and theoretically saturated [51] Another obvious limitation of the present study is that it does not describe whether the nature experiences in the programme had deeper meaning for participants in support of their health and well-being. Results regarding perceived health changes of participants under the course of the study will be described in another manuscript that is currently in preparation. Another limitation is that the WAYA programme was specifically developed for the Scandinavian countries and therefore cannot be directly transferred and implemented in other countries due to differences in nature settings, culture, and public health care services. However, the authors are of the opinion that the rigorous methodology by which the WAYA programme was developed can be used by public health care workers and outdoor facilitators in other countries, also those countries that are less developed and with lower income.

## 6. Recommendation for Practice and Further Research

Based on the results from this study it is recommended that wilderness/outdoor programmes allow for the inclusion of a large diversity of AYA cancer survivors. The variety in stage of survival, treatment, and disability of the participants was perceived as positive for group bonding and learning purposes. This requires that programmes include a good balance between group and individually adapted activities. Furthermore, a wilderness expedition for AYA cancer survivors requires that there be at least one alternative route to reach each overnight camping site. It is recommended that such alternative routes are offered to all participants, not just to those with obvious disabilities. Alternative routes should not only include shorter hiking distances, but also other ways of transport through the wilderness, such as kayaking, canoeing, or biking.

Mindfulness-based practices do not seem to be a regular part of adventure and wilderness programmes targeted at AYA cancer survivors [24]. Since mindfulness exercises seemed to support group cohesion, relaxation, and connection with nature in the present study, it is recommended that they are standardly incorporated. This is supported by the literature. Two systematic reviews have previously demonstrated that mindfulness-based interventions may decrease pain severity, anxiety and depression, and increase health-related quality of life of adult cancer patients [52,53]. A mindfulness-based intervention has also been shown to significantly reduce emotional distress and improve quality of life in AYA cancer survivors [54]. Although these reviews did not specifically address mindfulness-based interventions within a nature setting, another systematic review demonstrated that mindfulness practices in combination with exposure to wilderness may have synergistic positive effects on health [55]. In the WAYA programme, the mindfulness-based exercises included those that were more formal and guided, such as forest bathing, as well as informal exercises that occurred spontaneously, such as silent kayaking. It is of great interest to investigate which of these mindfulness-based exercises are most suited to the wilderness setting and supportive of the health and well-being of AYA cancer survivors. Specifically in relation to the self-realization process, as described in Næss’s ecosophy [27], further exploration of how AYA cancer survivors relate to nature and how they perceive these experiences to be supportive of their health and well-being is warranted. Photovoice, a methodology that facilitates the expression of participant experiences through photography and group discussion [56], could be a suitable method for this. Regarding the role of the facilitators in the mindfulness-based practices, it is recommended that they be active participants and share their own experiences and emotions rather than simply facilitating the exercises. The critical importance of facilitators in modelling openness, vulnerability, compassionate listening without judgment, and connection through mindfulness practices has previously been addressed [57]. To create a safe environment in the WAYA programme, it is recommended to more explicitly state and repeat to participants and facilitators that participation in—and sharing after—the mindfulness exercises is voluntary, and that all can pass if they not feel like sharing.

Based on the present findings with the WAYA programme, it is recommended that other wilderness programmes targeted to AYA cancer survivors also include both an expedition and a base-camp experience. Different types of settings seem to support participants in different ways. During a wilderness expedition, participants not only learn the practical skills of backpacking and setting up a camp site, but also mentally and even existentially learn and experience what it is to carry all you need to survive on your back, to adapt to nature and weather conditions, and to literally take a journey. During base-camp, more in-depth outdoor skills are practiced, in support of building a sense of independence among AYA cancer survivors and the development of their own personal outdoor practice. The present study did not specifically evaluate the best order of settings, i.e., first the expedition followed by base-camp or vice versa. It is also not known at present how each of these settings have contributed to the actual outdoor practice of AYA cancers survivors in the long-term. Further studies to investigate whether and how the WAYA programme elements contributed to the incorporation of outdoor activities in the daily lives of AYA cancer survivors are thus recommended.

## 7. Conclusions

Filling the apparent gap in the literature, the present study gives a detailed and systematic description of the development of a wilderness programme specifically targeted and adapted to the individual needs of AYA cancer survivors. Qualitative evaluation of the programme through focus group and individual interviews demonstrated that the WAYA programme is safe and well accepted. Study findings point to the added value of including a diverse group of AYA cancer survivors for group bonding and learning purposes. It was found that the inclusion of challenging activities and mindfulness-based exercises contribute to group bonding and cohesion. Furthermore, including both an expedition and base-camp component in the programme was perceived to be beneficial in supporting development of participants’ own personal outdoor practices. Recommendations from facilitators and AYA cancer survivors for optimization of the WAYA programme were mostly related to practical and logistical matters. These included to more slowly build up the (hiking) pace in the expedition, to improve the warmth of sleeping bags, variation in food, and offering sufficient alternative routes in the expedition portion of the programme to allow participants to choose the best option for them. The next steps in the study are to investigate the perceived impact of the WAYA programme on the health and well-being of AYA cancer survivors and evaluate the feasibility of performing a randomized controlled trial on the programme among this target group.

## Figures and Tables

**Table 1:** Demographic characteristics and competences of facilitators (*n* = 15).

Characteristics	Value
Age, mean ± SD	46.3 ± 8.4
Gender, n/%	
Male	7/46.7
Female	8/53.3
Nationality, n/%	
Sweden	7/46.7
Netherlands	3/20.0
USA	3/20.0
Finland	1/6.7
Norway	1/6.7
Competence/skills, n/%	
Outdoor/nature guide	8/47.1
Research data collection/physical measurements	5/33.3
Counselling/supervision (youth) groups	5/33.3
Survival training/instructor	4/26.7
Mindfulness-based practices	3/20.0
Kayak instructor	2/13.3
Nursing	2/13.3
Climbing instructor	1/13.3

**Table 2:** Demographic characteristics of study participants (*n* = 17).

Characteristics	Value
Age, mean ± SD	29.1 ± 3.8
Gender, n/%	
Male	6/35.3
Female	11/64.7
Ethnicity, n/%	
One or both parents from Sweden	16/94.1
Both parents from outside Sweden	1/5.9
Marital status, n/%	
Married-partner	8/47.1
Single	9/52.9
Family situation, n/%	
No children	16/94.1
Children	1/5.9
Education, n/%	
Comprehensive	1/5.9
High school	5/29.4
College/university	9/52.9
Other	2/11.8
Employment status, n/%	
Full time work	7/41.2
Part time work	4/23.5
Full time education	2/11.8
Unable to work	4/23.5
Economic situation, n/%	
(how often do you have good finances to be able to do the same things as your friends)	
Always	7/41.2
Often	8/47.1
Sometimes	1/5.9
Seldom	1/5.9
Primary cancer type, n/%	
Haematological	6/35.3
Brain	5/29.4
Testicular	2/11.8
Thyroid	2/11.8
Osteosarcoma	1/5.9
Breast	1/5.9
Age at cancer diagnosis, mean ± SD (min-max)	18.9 ± 8.5 (1.5–29)
Time since last treatment, n/%	
<3 months	4/23.5
4–11 months	1/5.9
1–5 years	4/23.5
>5 years	7/41.2
On active treatment	1/5.9

**Table 3:** Health issues reported by participants (*n* = 17).

Health Issue	n/%
Pain (all pain)	12/70.6
Mental health problems (all combined)	10/58.8
Allergies/asthma	6/35.3
Cognitive dysfunction	6/35.3
Depression	5/29.4
Gastro-intestinal problems	4/23.5
Anxiety	4/23.5
Visual impairment	2/11.8
Hearing compromised	2/11.8
Sleeping problems	2/11.8
Balance problems	2/11.8
Hormonal dysfunction	2/11.8
Tiredness (general)	1/5.9
Concentration problems	1/5.9
Memory problems	1/5.9
Skin sensitivity	1/5.9
Restricted joint movement	1/5.9
Paresis (partial)	1/5.9
Gout	1/5.9
Osteoporosis	1/5.9
Anaemia	1/5.9

**Table 4:** Structure of the WAYA Programme.

Characteristics	Programme Model		
	Expedition	Online Coaching	Base-Camp
Duration (days)	8	90	4
Group size	10	NA **	10
Group structure	closed	NA	closed
Amount of time in group activities * (h)	4–8	NA	4–6
Amount of time in structured activities * (h)	6–8	NA	4–6
Amount of free time * (h)	3	NA	6
Facilitator-participant ratio	1:2	1:1	1:3

* Average per day, where a day was counted from 7:30 to 21:00; ** NA = not applicable.

**Table 5:** Individual adaptation of the WAYA programme activities.

Programme Activity	Disability of Participant (n)	Adaptation/Alternative
Hiking trails	Peripheral neuropathy (n = 1)	Kayaking, shorter hiking trails, extra breaks, warm water bottles for pain relief
	Restricted joint movement (n = 1)	Shorter hiking trails, hiking with Nordic hiking poles, extra breaks, canoeing
	Visual impairments (n = 2)	Assistance by facilitators, boat transport, canoeing, swimming to shore with guidance of facilitator
	Obsessive-compulsive disorder symptoms (n = 1)	Guidance by facilitator to assure that nothing is left behind on the trail
	Cognitive dysfunction (n = 1)	Shorter hiking trails, canoeing
	Paresis (n = 1) Balance problems (n = 2)	Hiking with Nordic hiking poles
Backpacking	Peripheral neuropathy (n = 1) Restricted joint movement (n = 1) Visual impairments (n = 2)	Backpack carried by facilitators
	Balance problems (n = 4) Pain (n = 4) Skin sensitivity (n = 1)	Bigger items such as tent carried by facilitators
Setting up/breaking down camp	Visual impairments (n = 1)	Long rope from the tent to trees in forest for guidance relating to going to the “bathroom” in the woods
	Cognitive dysfunction (n = 6) Concentration problems (n = 1)	Allowing extra time to sleep in the morning and for breaking down camp and packing up, full-time assistance by facilitator throughout all camping activities
	Obsessive-compulsive disorder symptoms (n = 1)	Allowing extra time for breaking down camp and packing up. Check by facilitators that all outdoor equipment is in order and in place in the tent
	Anxiety/depression (n = 9)	Supportive talks, individually guided meditation exercises, allowing extra time by themselves to process and recover, journaling
Kayaking	Visual impairments (n = 2)	Double kayak with facilitator
Rock climbing	Visual impairments (n = 2) Peripheral neuropathy (n = 1)	Alternative activities such as learning bushcraft skills
Eating/Cooking	Allergies/intolerance (n = 6)	Home-made freeze-dried food
	Gastro-intestinal problems (n = 4)	Medication

## Data Availability

Detailed reports concerning underlying data of this study can be requested from the corresponding author.

## Supplementary Materials

The following supporting information can be downloaded at: https://www.mdpi.com/article/10.3390/ijerph191912012/s1, File S1: Field diary, File S2: Risk & safety plan, File S3: Focus group interview guide, File S4: Interview guide.
