# Supplementary material for: The Development and Acceptability of a Wilderness Programme to Support the Health and Well-Being of Adolescent and Young Adult Cancer Survivors: The WAYA Programme
Source: Int J Environ Res Public Health. 2022 Sep 22;19(19):12012. doi: 10.3390/ijerph191912012 (PMC9566286; doi:10.3390/ijerph191912012)
Supplement: Supplementary file 1 [file ijerph-19-12012-s001.zip › supplementary/Supplementary file S1 Field diary.pdf]

NAFKAM | Nasjonalt forskningssenter innen komplementær og alternativ medisin

Mittuniversitetet

MID SWEDEN UNIVERSITY

Field diary Facilitators

Wilderness Program 14-21 June 2021

The **WAYA** study (Wilderness program for Adolescent and Young Adult cancer survivors)

Contact list responsible body

Name	Role	Responsibility
Mats Jong	Principal Investigator (PI)	WAYA study research activities
Miek Jong	Co-Principal Investigator (CPI)	WAYA study research coordination
Sture Espwal	Leader Department of Health Sciences, Mid Sweden University	WAYA study, as part of research project at the Department of Health Sciences

Relevant emergency telephone numbers

Telephone	Body	Website
**112**	Räddningstjänsten	https://www.sosalarm.se/112/Om-112/Att-larma-112/nar-ska-du-ringa-112/
1177	Sjukvårdsupplysningen	http://www.1177.se/
0200 290090	Sjöräddningen, förebyggande utryckning	https://www.sjoraddning.se/om-oss/kontakta-oss
054 22 12 00	Study Insurance Kammarkollegiet	https://www.kammarkollegiet.se/vara-tjanster/forsakring-och-riskhantering/hitta-forsakringsprodukt/personforsakringar/ovriga-personskador/sarskilt-personskadeskydd

PROGRAM

Day 1: 14 June 2021

**Location**	Glosand (Härnösand): Coordinates N 6933196, E 646543 (SWEREF 99 TM)
**Journey**	Home address till Glosand by plane, train, bus, car, foot
**Starting time**	As from 14:00
**Measurements**	- COVID-19 tests<br>- Blood Pressure and Heart Rate<br>- Height, weight<br>- Six-minute walk test<br>- Oxygen Uptake (OU)
**Instructions**	- Delivery of outdoor equipment and how to use it (preparing for the night)<br>- Pooping, hygiene<br>- Cooking/food/water instructions
**Activities**	- Start of the Program: introduction and rules<br>- Setting up tent<br>- Outdoor cooking<br>- Camp fire
**Exercises**	- Closing mindfulness meditation

Day 1: 14 June 2021

Have you observed any 'almost' accidents/incidents/close call:

☐ YES ☐ NO

in case of YES, please describe what:

Have any accidents/incidents occurred today?

☐ YES ☐ NO

in case of YES, please describe what, where, how:

Name of the participant(s) involved:

Experienced effect on the participants (describe condition, symptoms, etc):

Intensity of the accident/incident (neglectable, mild, noticeable, severe):

Action/measure (describe how you/others have acted such as care, medication, other):

Follow-up (describe if and what is needed regarding follow-up monitoring):

Have you provided any medication to participants (other than related to the accident/incident)?

☐ YES ☐ NO

in case of YES, please describe what:

Have you provided any specific support to participants (physical, mental, other)?

☐ YES ☐ NO

in case of YES, please describe what:

Have you given any specific program-related instruction to participants?

☐ YES ☐ NO

in case of YES, please describe what:

Did you lack any outdoor equipment or miss any other necessary items?

☐ YES ☐ NO

in case of YES, please describe what:

Please estimate the number of hours today (between 7:30-21:00) in which participants had free (leisure) time, and thus were not involved in any program-related (group) activity

Number: ….. hours

Other relevant notes:

Day 2: 15 June 2021

**Location**	Dalsjön (Käxed, Kramfors): Coordinates N 7000417, E 672355 (SWEREF 99 TM)
**Journey**	Driving: 1:15 hour by car<br>Distance: 7 km hiking (6 hours) to Dalsjön
**Starting time**	As from 7:30
**Measurements**<br>(if not finished the day before)	- COVID-19 tests<br>- Blood Pressure and Heart Rate<br>- Height, weight<br>- Six-minute walk test<br>- Oxygen Uptake (OU)
**Instructions**	- Packing-up (back-pack)<br>- Mapping/compass<br>- Safety skills training<br>- Equipment planning<br>- Eatable things in nature<br>- Allemansrätten and leave no trace
**Activities**	- Hiking (including lunch)<br>- Setting up camp (dinner)
**Exercises**	- Check-in (before leaving)<br>- Check-out: (end of the day)<br>- Reflective mindfulness exercise

Day 2: 15 June 2021

Have you observed any ‘almost’ accidents/incidents/close call:

☐ YES ☐ NO

in case of YES, please describe what:

Have any accidents/incidents occurred today?

☐ YES ☐ NO

in case of YES, please describe what, where, how:

Name of the participant(s) involved:

Experienced effect on the participants (describe condition, symptoms, etc):

Intensity of the accident/incident (neglectable, mild, noticeable, severe):

Action/measure (describe how you/others have acted such as care, medication, other):

Follow-up (describe if and what is needed regarding follow-up monitoring):

Have you provided any medication to participants (other than related to the accident/incident)?

☐ YES ☐ NO

in case of YES, please describe what:

Have you provided any specific support to participants (physical, mental, other)?

☐ YES ☐ NO

in case of YES, please describe what:

Have you given any specific program-related instruction to participants?

☐ YES ☐ NO

in case of YES, please describe what:

Did you lack any outdoor equipment or miss any other necessary items?

☐ YES ☐ NO

in case of YES, please describe what:

Please estimate the number of hours today (between 7:30-21:00) in which participants had free (leisure) time, and thus were not involved in any program-related (group) activity

Number: …..hours

Other relevant notes:

Day 3: 16 June 2021

**Location**	Kälaviken (Käl, Kramfors): Coordinates N 6999825, E 677273 (SWEREF 99 TM)
**Journey**	Distance: 10 km (8 hours) hiking to Kälaviken
**Starting time**	As from 7:30
**Instructions**	- Mapping/compass<br>- Safety skills training<br>- Eatable things in nature<br>- Allemansrätten and leave no trace
**Activities**	- Hiking (including lunch)<br>- Setting up camp (dinner)
**Exercises**	- Check-in (before leaving)<br>- Check-out: (end of the day)<br>- Reflective mindfulness exercise

Day 3: 16 June 2021

Have you observed any ‘almost’ accidents/incidents/close call:

☐ YES ☐ NO

in case of YES, please describe what:

Have any accidents/incidents occurred today?

☐ YES ☐ NO

in case of YES, please describe what, where, how:

Name of the participant(s) involved:

Experienced effect on the participants (describe condition, symptoms, etc):

Intensity of the accident/incident (neglectable, mild, noticeable, severe):

Action/measure (describe how you/others have acted such as care, medication, other):

Follow-up (describe if and what is needed regarding follow-up monitoring):

Have you provided any medication to participants (other than related to the accident/incident)?

☐ YES ☐ NO

in case of YES, please describe what:

Have you provided any specific support to participants (physical, mental, other)?

☐ YES ☐ NO

in case of YES, please describe what:

Day 3: Have you given any specific program-related instruction to participants?

☐ YES ☐ NO

in case of YES, please describe what:

Did you lack any outdoor equipment or miss any other necessary items?

☐ YES ☐ NO

in case of YES, please describe what:

Please estimate the number of hours today (between 7:30-21:00) in which participants had free (leisure) time, and thus were not involved in any program-related (group) activity

Number: …..hours

Other relevant notes:

Day 4: 17 June 2021

**Location**	Kälaviken (Käl, Kramfors): Coordinates N 6999825, E 677273 (SWEREF 99 TM)
**Journey**	Kayaking in Kälaviken bay (2-3 hours)
**Starting time**	As from 7:30
**Instructions**	- Mapping/compass<br>- Safety skills training<br>- Eatable things in nature<br>- Allemansrätten and leave no trace<br>- Kayaking safety & skills
**Activities**	- Kayaking
**Exercises**	- Check-in (before leaving)<br>- Check-out: (end of the day)<br>- Meditation

Day 4: 17 June 2021

Have you observed any ‘almost’ accidents/incidents/close call:

☐ YES ☐ NO

in case of YES, please describe what:

Have any accidents/incidents occurred today?

☐ YES ☐ NO

in case of YES, please describe what, where, how:

Name of the participant(s) involved:

Experienced effect on the participants (describe condition, symptoms, etc):

Intensity of the accident/incident (neglectable, mild, noticeable, severe):

Action/measure (describe how you/others have acted such as care, medication, other):

Follow-up (describe if and what is needed regarding follow-up monitoring):

Have you provided any medication to participants (other than related to the accident/incident)?

☐ YES ☐ NO

in case of YES, please describe what:

Have you provided any specific support to participants (physical, mental, other)?

☐ YES ☐ NO

in case of YES, please describe what:

Have you given any specific program-related instruction to participants?

☐ YES ☐ NO

in case of YES, please describe what:

Did you lack any outdoor equipment or miss any other necessary items?

☐ YES ☐ NO

in case of YES, please describe what:

Please estimate the number of hours today (between 7:30-21:00) in which participants had free (leisure) time, and thus were not involved in any program-related (group) activity

Number: …..hours

Other relevant notes:

Day 5: 18 June 2021

**Location**	Baggsandsbukten (Mjältön, Kramfors): Coordinates N 6994059, E 677999 (SWEREF 99 TM)
**Journey**	Distance: Kayaking (6 km)<br>Hiking (2.5 km)
**Starting time**	As from 7:30
**Instructions**	- Mapping/compass<br>- Safety skills training<br>- Eatable things in nature<br>- Allemansrätten and leave no trace<br>- Kayaking safety & skills
**Activities**	- Kayaking (including lunch)<br>- Hiking<br>- Setting up camp (dinner)
**Exercises**	- Check-in (before leaving)<br>- Check-out: (end of the day)<br>- Mindfulness exercise

Day 5: 18 June 2021

Have you observed any ‘almost’ accidents/incidents/close call:

☐ YES ☐ NO

in case of YES, please describe what:

Have any accidents/incidents occurred today?

☐ YES ☐ NO

in case of YES, please describe what, where, how:

Name of the participant(s) involved:

Experienced effect on the participants (describe condition, symptoms, etc):

Intensity of the accident/incident (neglectable, mild, noticeable, severe):

Action/measure (describe how you/others have acted such as care, medication, other):

Follow-up (describe if and what is needed regarding follow-up monitoring):

Have you provided any medication to participants (other than related to the accident/incident)?

☐ YES ☐ NO

in case of YES, please describe what:

Have you provided any specific support to participants (physical, mental, other)?

☐ YES ☐ NO

in case of YES, please describe what:

Day 5: Have you given any specific program-related instruction to participants?

☐ YES ☐ NO
in case of YES, please describe what:

Did you lack any outdoor equipment or miss any other necessary items?

☐ YES ☐ NO
in case of YES, please describe what:

- Please estimate the number of hours today (between 7:30-21:00) in which participants had free (leisure) time, and thus were not involved in any program-related (group) activity

  Number: …..hours

Other relevant notes:

Day 6: 19 June 2021

**Location**	Baggsandsbukten (Mjältön, Kramfors): Coordinates N 6994059, E 677999 (SWEREF 99 TM)
**Journey**	Distance: Hiking (6 km) to top Mjältön island
**Starting time**	As from 7:30
**Instructions**	- Mapping/compass<br>- Safety skills training<br>- Eatable things in nature<br>- Allemansrätten and leave no trace
**Activities**	- Hiking<br>- Gorge climbing
**Exercises**	- Check-in (before leaving)<br>- Check-out: (end of the day)<br>- Mindfulness exercises

Day 6: 19 June 2021

Have you observed any ‘almost’ accidents/incidents/close call:

☐ YES ☐ NO

in case of YES, please describe what:

Have any accidents/incidents occurred today?

☐ YES ☐ NO

in case of YES, please describe what, where, how:

Name of the participant(s) involved:

Experienced effect on the participants (describe condition, symptoms, etc):

Intensity of the accident/incident (neglectable, mild, noticeable, severe):

Action/measure (describe how you/others have acted such as care, medication, other):

Follow-up (describe if and what is needed regarding follow-up monitoring):

Have you provided any medication to participants (other than related to the accident/incident)?

☐ YES ☐ NO
in case of YES, please describe what:

Have you provided any specific support to participants (physical, mental, other)?

☐ YES ☐ NO
in case of YES, please describe what:

Have you given any specific program-related instruction to participants?

☐ YES ☐ NO

in case of YES, please describe what:

Did you lack any outdoor equipment or miss any other necessary items?

☐ YES ☐ NO

in case of YES, please describe what:

Please estimate the number of hours today (between 7:30-21:00) in which participants had free (leisure) time, and thus were not involved in any program-related (group) activity

Number: …..hours

Other relevant notes:

Day 7: 20 June 2021

**Location**	Sund (Kramfors): Coordinates N 6995006, E 674620 (SWEREF 99 TM)
**Journey**	Distance: Hiking (4 km)<br>Kayaking (1.5 km)
**Starting time**	As from 7:30
**Instructions**	- Mapping/compass<br>- Safety skills training<br>- Eatable things in nature<br>- Allemansrätten and leave no trace<br>- Kayaking instructions
**Activities**	- Hiking<br>- Kayaking
**Exercises**	- Check-in (before leaving)<br>- Check-out (end of the day)

Day 7: 20 June 2021

Have you observed any 'almost' accidents/incidents/close call:

☐ YES ☐ NO

in case of YES, please describe what:

Have any accidents/incidents occurred today?

☐ YES ☐ NO

in case of YES, please describe what, where, how:

Name of the participant(s) involved:

Experienced effect on the participants (describe condition, symptoms, etc):

Intensity of the accident/incident (neglectable, mild, noticeable, severe):

Action/measure (describe how you/others have acted such as care, medication, other):

Follow-up (describe if and what is needed regarding follow-up monitoring):

Have you provided any medication to participants (other than related to the accident/incident)?

☐ YES ☐ NO

in case of YES, please describe what:

Have you provided any specific support to participants (physical, mental, other)?

☐ YES ☐ NO

in case of YES, please describe what:

Have you given any specific program-related instruction to participants?

☐ YES ☐ NO

in case of YES, please describe what:

Did you lack any outdoor equipment or miss any other necessary items?

☐ YES ☐ NO

in case of YES, please describe what:

Please estimate the number of hours today (between 7:30-21:00) in which participants had free (leisure) time, and thus were not involved in any program-related (group) activity

Number: …..hours

Other relevant notes:

Day 8: 21 June 2021

**Location**	Sund (Kramfors): Coordinates N 6995006, E 674620 (SWEREF 99 TM)
**Journey**	Travelling home by car, train, bus, flight
**Starting time**	As from 7:30
**Instructions**	- Packing-up for home
**Exercises**	- Check-out (before leaving)

Day 8: 21 June 2021

Have you observed any ‘almost’ accidents/incidents/close call:

☐ YES ☐ NO

in case of YES, please describe what:

Have any accidents/incidents occurred today?

☐ YES ☐ NO

in case of YES, please describe what, where, how:

Name of the participant(s) involved:

Experienced effect on the participants (describe condition, symptoms, etc):

Intensity of the accident/incident (neglectable, mild, noticeable, severe):

Action/measure (describe how you/others have acted such as care, medication, other):

Follow-up (describe if and what is needed regarding follow-up monitoring):

Have you provided any medication to participants (other than related to the accident/incident)?

☐ YES ☐ NO

in case of YES, please describe what:

Have you provided any specific support to participants (physical, mental, other)?

☐ YES ☐ NO

in case of YES, please describe what:

Have you given any specific program-related instruction to participants?

☐ YES ☐ NO

in case of YES, please describe what:

Did you lack any outdoor equipment or miss any other necessary items?

☐ YES ☐ NO

in case of YES, please describe what:

Please estimate the number of hours today (between 7:30-21:00) in which participants had free (leisure) time, and thus were not involved in any program-related (group) activity

Number: …..hours

Other relevant notes:

Telephone numbers of facilitators
