# Supplementary material for: The Development and Acceptability of a Wilderness Programme to Support the Health and Well-Being of Adolescent and Young Adult Cancer Survivors: The WAYA Programme
Source: Int J Environ Res Public Health. 2022 Sep 22;19(19):12012. doi: 10.3390/ijerph191912012 (PMC9566286; doi:10.3390/ijerph191912012)
Supplement: Supplementary file 1 [file ijerph-19-12012-s001.zip › supplementary/Supplementary file S2 Risk & Safety Plan.pdf]

Risk & Safety Plan:

The WAYA study (Wilderness program for Adolescent and Young Adult cancer survivors)

Mid Sweden University & NAFKAM at the Artic University of Norway

May 2021

Authors:
Miek Jong
Mats Jong

Table of Contents

Risk & Safety Plan: ... 1

1. Risk and safety Plan of the WAYA study ... 3

1.1 Objective of the Risk and Safety Plan ... 3

1.2 Overview of the WAYA study ... 3

2 Contact list responsible body ... 3

3 Relevant emergency telephone numbers ... 4

4 Facilitators ... 4

4.1 Group 1 Wilderness Program: 14-21 June 2021 ... 4

4.2 Group 2 Wilderness program: 27 June – 4 July 2021 ... 4

4.3 Group 3 Holiday program: 5-12 July 2021 ... 4

4.4 Group 4 Holiday program: 15-22 July 2021 ... 4

5 Description of the wilderness area ... 4

5.1 Terrain ... 4

5.2 Weather ... 5

5.3 Cell phone coverage ... 5

6 General description of the participants ... 6

7 Safety measures ... 6

7.1 Competent staff ... 6

7.2 COVID-19 testing ... 7

7.3 Accidents/incidents ... 7

7.4 Emergency procedures and evacuation ... 7

8 Description of activities in the wilderness program ... 8

9 Risk analysis for the study ... 9

9.1 Risk exposure/consequence matrix ... 9

9.2 Risk exposure/consequence matrix of the WAYA study ... 11

10 Equipment list related to the risks identified ... 15

11 Safety Instructions participants ... 16

12 Safety monitoring ... 17

13 References ... 17

14 Appendix I: Emergency and Evacuation procedures ... 18

1. Risk and safety Plan of the WAYA study

1.1 Objective of the Risk and Safety Plan

The overall objective with this Risk and Safety Plan is:

- To assess potential risks that can be predicted
- Take necessary precautions and preparations to perform the interventions with acceptable risks
- Have well communicated practices in case of an emergency
- To ensure a safe evacuation if needed

1.2 Overview of the WAYA study

**Study Name**	WAYA study	
**Responsible Body**	Mid Sweden University	
**Website of the study**	https://www.miun.se/Forskning/forskningsprojekt/pagaende-forskningsprojekt/waya/waya---information/	
**Intervention**	**Wilderness Program**	**Holiday Program (Control)**
**Number of participants**	20-25	20-25
**Date intervention program**	14-21 June 2021<br>and<br>27 June – 4 July 2021	5-12 July 2021<br>and<br>15-22 July 2021
**Date follow-up program**	10-13 September 2021<br>and<br>17-20 September 2021	24-27 September 2021<br>and<br>1-4 October 2021
**Place of the intervention**	Skuleskogen, Höga Kusten	Hotell Södra Berget, Sundsvall
**Age participants (years)**	16-39	16-39

2 Contact list responsible body

**Name**	**Role**	**Responsibility**
Mats Jong	Principal Investigator (PI)	WAYA study research activities
Miek Jong	Co-Principal Investigator (CPI)	WAYA study research coordination
Sture Espwal	Leader Department of Health Sciences, Mid Sweden University	WAYA study, as part of research project at the Department of Health Sciences

3 Relevant emergency telephone numbers

Telephone	Body	Website
112	Räddningstjänsten	https://www.sosalarm.se/112/Om-112/Att-larma-112/nar-ska-du-ringa-112/
1177	Sjukvårdsupplysningen	http://www.1177.se/
0200 290090	Sjöräddningen, förebyggande utryckning	https://www.sjoraddning.se/om-oss/kontakta-oss
054 22 12 00	Study Insurance Kammarkollegiet	https://www.kammarkollegiet.se/vara-tjanster/forsakring-och-riskhantering/hitta-forsakringsprodukt/personforsakringar/ovriga-personskador/sarskilt-personskadeskydd

4 Facilitators

4.1 Group 1 Wilderness Program: 14-21 June 2021

Name	Role	Specific Tasks/Competences	Relevant education and training

4.2 Group 2 Wilderness program: 27 June – 4 July 2021

Name	Role	Specific Tasks/Competences	Relevant education/training

4.3 Group 3 Holiday program: 5-12 July 2021

Name	Role	Specific Tasks/Competences	Relevant education/training

4.4 Group 4 Holiday program: 15-22 July 2021

Name	Role	Specific Tasks/Competences	Relevant education/training

5 Description of the wilderness area

5.1 Terrain

The wilderness program takes place in the area around (south-west, south) Skuleskogen National Park and the island Mjältön (Höga Kusten).

The hiking trails will cover the following terrain:
- forest paths with crossing streams, tree roots, fallen trees, and branches that stick out
- rocky paths with (loose) stones
- rocky paths with wet (slippery) water parts
- paths along wetland (marsh vegetation) and lakes

- paths to the top of Mjältön island (236 meters high) that requires to make use of the hands, and kneeling or sit down when descending
- cross-terrain hike with soft moss forest grounds, bushes, stones, along swamps and crossing streams

The camping sites will cover the following terrain:
- Peninsula (163 meters high), along marshes and a lake surrounded by boreal taiga forest, with low bushes, grass and stones on the ground.
- forest directly by the sea, with thick moss on the ground
- open forest area along the beach, with low bushes, grass and stones on the ground
- open grass field by the sea

The sea kayak trips will cover the following terrain:
- shallow sea bay water close to the shore (1-2 meters deep) with a temperature of +10-20°C (dependent on current weather conditions; strong wind currents can cause water upwelling that might shift the upper water layer temperature from +20°C to +5°C within hours)
- sea bay crossing through deeper water (500-1000 meters deep, +10-20°C)
- sandy beaches for getting in and out the kayak
- shoreline with (loose) stones for getting in and out the kayak

5.2 Weather

The weather can vary greatly from one day to the next, or even within the same day. It can be sunny, cloudy, cool, windy, raining and sometimes even some light snow has been reported.
The air temperature during the day is expected to vary from +10 to +25°C degrees, but at night the temperature can drop down to +1 or +2°C degrees.
The water temperature in the sea bay can vary from +10-20°C. The seawater temperature is dependent on current weather conditions, strong wind currents can cause water upwelling that might shift the upper water layer temperature from 20°C to 5°C within hours.
Long term weather forecasts are not reliable in the area. Predominant wind directions during the summer season are onshore winds during daytime and offshore during nights. Besides the on-offshore wind effect is a northwest wind dominant. The wind is often calm in early morning and late evenings. Specifically, wind from the north, north-east will affect the kayaking and may cause (bigger) waves.

5.3 Cell phone coverage

There is sufficient cell phone coverage during most of the hiking and kayaking trips and camping sites. At some places the internet works slower than at other places. During the 3rd day of the wilderness program (16 June and 29 June 2021), there is no cellphone coverage for a distance of 4 km along the hike from Dalsjön to Kälaviken in Skuleskogen national park. The marked hiking path follows a stream valley that is situated in cellphone shadow area, in most cases there are possibilities to find sufficient coverage on adjacent high ground terrain.

The staff will have smartphones with SOS -alarm application installed. The application has many useful features as a direct dial that supports the SOS-alarm central with the caller's current location via GPS. The user will be notified if any accidents (i.e. forest fires) occur in the near situated area.

6 General description of the participants

The participants are adolescent and young adult cancer survivors. The age is 16-39 years (most between 25-30 years). Participants are of any sex (>75% is women). Participants have been diagnosed with any type of cancer (brain tumor, breast cancer, thyroid cancer, ovarian cancer, leukemia, etc). They are (recently) out of cancer treatment. Some participants still receive cancer-related (medical) treatments. All participants have good understanding of the Swedish language.
All participants have the ability to walk 2 km without pausing, but they differ greatly with respect to their physical and mental condition. Some participants need aids in order to walk 2 km without pausing such as Nordic walking sticks, a support dog, or a supporting arm.
Some participants are physically quite fit and have been out in nature before, whereas others have very little to no experience with being out in nature. Several participants have sight, hearing, or balance problems. Several participants have cognitive fatigue, concentration problems or depressive symptoms and cannot take up too much information. Several participants have food allergies and special diet wishes (vegan, vegetarian, gluten-free) or an allergy for perfumes.

7 Safety measures

All participants and facilitators will be insured under the intervention period by a specific study insurance via Kammarkollegiet as paid for by Mid Sweden University. (https://www.kammarkollegiet.se/vara-tjanster/forsakring-och-riskhantering/hitta-forsakringsprodukt/personforsakringar/ovriga-personskador/sarskilt-personskadeskydd)

Mats Jong, Principal Investigator (PI) in the study, will monitor the weather conditions and respond accordingly to imminent threats that may lead to potential accidents or incidents. The PI will decide to relocation of a group, to choose alternative routes or camping sites, to get additional needed resources/equipment, to evacuate the group or to end the program if needed in case of weather conditions, group/personal conditions or in cases of other emergencies.

Miek Jong, Co-Principal Investigator (CPI), has a list of contact persons of participants and will contact them in case of emergencies.

7.1 Competent staff

Wilderness program:
A total of 10-13 participants will be guided by 6-7 facilitators (2:1 ratio participants/facilitator).
Facilitators have competences related to:
- basic first-aid in field conditions;
- instructions in safety procedures related to outdoor events and proper equipment usage (fuel, fire, life protection);
- instruction in emergency procedures like medical, evacuation, weather, or fire;
- navigation skills (map and compass use for navigation);
- counseling, teaching, supervisory skills, leadership, communication, relational skills;
- sea kayaking and climbing instruction skills;
- managing, preparing, and conserving water, food, and shelter;
- wilderness survival, expedition and environmental conservation skills;

- group management skills (containment, control, safety, conflict resolution, behavior management);
- instruction skills related to sanitation procedures (water, waste, food, pooping/peeing, field hygiene);
- research skills, report writing, including development and maintenance of logs and case report forms:
- knowledge of relevant national, regional and local guidelines and regulations.

Holiday Program:
A total of 10-13 participants will be supervised by 2 facilitators (6:1 ratio participants/facilitator). The two facilitators of the holiday program have the necessary supervision competences related to group management, counseling, teaching, leadership, communication, and relational skills.

7.2 COVID-19 testing

Upon arrival at the first intervention day, participants and facilitators in the wilderness and holiday programs will be tested for COVID-19 by means of a Rapid Response™ COVID-19 Antigen Rapid Test Cassette from BioServ UK. This test has a sensitivity of 95%, and a specificity of 100%. The tests will be performed by qualified personnel according to a validated instruction protocol from Labex Scandinavia (https://labex.com/wp-content/uploads/BioServUK-Saliva-Ag-Kit-Factsheet-V1.1.pdf). COVID-19 testing of all participants and facilitators will be repeated in the morning of the second day of the interventions in order to minimize the risk of false negatives. During the ongoing program further COVID-19 testing will be performed in case a participant or facilitator exhibits COVID-19 related symptoms, initially with the Antigen test and if positive with a PCR test through Region Västernorrland (see also Chapter 9).

7.3 Accidents/incidents

The safety of wilderness-type programs for young cancer survivors has been little investigated so far. A recent review reported that only two out of 15 studies (13%) on wilderness programs reported on the occurrence of adverse events and safety of wilderness programs for young cancer survivors (Jong et al). Accidents/incidents that were reported were: insect bites, abrasions, sore throats, headaches, upset stomachs, sliver in the finger, laceration of the head of a facilitator, other minor injuries, and anxiety.

Despite little investigation, the following accidents/incidents have been assessed to be of risk of occurring during the study interventions: COVID-19 infection, sunburn, wound, sprain, blister, sore, nausea, dizziness, headache, hypothermia, constipation, insomnia, allergic reaction (including anaphylactic shock), fracture, larger wound, mosquito bite, wasp sting, snake bite, tick, loss of necessary medication, cold, sore throat, stomachache, diarrhea, anxiety/fear, exhaustion, depression, brain fatigue, food poisoning, alcohol/drug abuse.

Read more on necessary actions regarding accidents/incidents in Chapter 9.

7.4 Emergency procedures and evacuation

Facilitators will act according to site-specific emergency and evacuation tables that are attached in Appendix I.

Evacuation is used as a general term for transporting someone from the intervention program. Evacuation can be needed in case of medical problems related to accidents/incidents, exhaustion, or COVID-19. However, evacuation can also be needed in case of mental health issues, a family emergency, or the assessment of the PI that the participants' behavior poses a threat to themselves or others in the group.

When assessing the need for an evacuation and type of evacuation, the PI will consider the following factors:
- The available resources on side (first aid)
- The type/seriousness of accident/incident
- The (medical) condition of the person
- Available equipment for evacuation
- Location of the person
- Trail and weather conditions

Wilderness program-related emergency evacuations:
About 90% of the hiking/kayaking tracks and camping sites are within 30-60 minutes access to a road for ambulance evacuation. Örnsköldsvik hospital (Sjukhusgatan 8, 891 35 Örnsköldsvik) can be reached by ambulance/car in less than 30 minutes from the nearest roads. Air evacuation via helicopter is available and can be used if an emergency needs immediate assistance and if the authorities evaluate it as necessary.

Holiday program-related emergency evacuations:
Hotell Södra Berget is within a 20 minutes' drive by car/ambulance from Sundsvall hospital (Lasarettsvägen 21, 856 43 Sundsvall).

8 Description of activities in the wilderness program

**Activity**	**Date (2021)**	**Description/Intensity**
**Wilderness program:**	14-21 June or<br>27 June - 4 July	The wilderness program has a duration of 8 days (7 nights)
Backpacking, hiking, equipment planning	15, 16, 18-20 June or 28, 29 June, 1-3 July	2-10 km hiking with backpacks of 2-15 kg according to each individual's capacity. The backpack will contain necessary outdoor equipment (clothes, tent, sleeping bag, matrass, cooking gear, first-aid kit, food, water bottles)
Sea kayaking	17, 18, 20 June or 30 June, 1 and 3 July	1.5 - 8 km of paddling, either in an own kayak or in a two-person kayak
Rock climbing	19 June or 2 July	20 meters moderate steep up along the left side of the gorge by means of security ropes
Camping, outdoor skills, cooking out, safety skills training, eatable things in nature, allmanrett and leave no trace	14-21 June or<br>27 June - 4 July	8 days being and eating out in nature with no access to a toilet/shower, and sleeping in a tent for 7 nights

Mapping/compass/orienting	15-20 June or<br>28 June – 3 July	Each of these days, two participants will take the lead in mapping and navigating the tracks/routes
Leisure activities: singing, bathing/swimming, fishing, campfire, reading, taking pictures, playing games/cards	14-21 June or<br>27 June - 4 July	Depending on each individual need and wishes
Reflective Practice exercises: reflective writing/journaling, mindfulness meditation, forest bathing, other guided and non-guided (reflective) meditations.	14-21 June or<br>27 June - 4 July	Reflective, mindfulness exercises with a duration varying from 5 minutes to 30 minutes.
**Holiday Program:**	5-12 July or<br>15-22 July	The holiday program has a duration of 8 days (7 nights)
Leisure activities: spa, bathing, swimming, watching movies, karaoke, sunbathing, fishing, reading, social media/phone, playing games/cards, bowling, eating	5-12 July or<br>15-22 July	Depending on each individual's needs and wishes. Breakfast, lunch and dinner will be together with the group. All other activities can be done by themselves or with others.

9 Risk analysis for the study

9.1 Risk exposure/consequence matrix

Potential risks are assessed and analyzed for acceptability according to the risk exposure/consequence matrix of the Swedish Mountain Society (Svenska Fjällklubben, accessed 29/04/21) as depicted below.

	**Consequence**			
**Likelihood**	Neglectable	Mild	Noticeable	Severe
Probable	4	8	12	16
Possible	3	6	9	12
Less likely	2	4	6	8
Unlikely	1	2	3	4

Risk factors 1-4 (green) acceptable risk; 6-8 (yellow) acceptable risk but requires close monitoring; 9-12 (orange) acceptable risk, but requires close monitoring to prevent the risk, follow-up, and

evacuation where necessary; 16 (red) not acceptable risk, should be prevented at any costs and in case it happens it requires immediate evacuation.

The likelihood that an event that causes a risk will occur is assessed by an estimate of how often it may occur during the intervention: 1. Unlikely - the risk is practically non-existent for the event to occur; 2. Less likely - the risk is small for the event to occur; 3. Possible - there is a risk that the event will occur; 4. Probable - it is very likely that the event will occur.

The consequence of an event that causes a risk is assessed through the impact that it may have on the participants. 1. Neglectable - small enough or unimportant enough to be neglected; 2. Mild - perceived as low/small impact; 3. Noticeable - perceived as troublesome; 4. Severe – serious impact.

A systematic stepwise approach will be used to manage risk and safety during the wilderness program. This approach aims to prevent accidents and create a comfortable and safe learning environment for all participants and staff. An example for a situation based systematic stepwise approach is given below. The approach is used to prevent a chain of reactions that might escalate.

Example of a stepwise approach to manage risk & safety during the wilderness program

**Situation**	**Action**
Information of field conditions. The weather might be rainy and cold despite it is summertime. Participants that usually get cold understand the importance of sufficient clothing	Written information in advance
Inform of routs, plans and weather forecast	Give verbal information in field. Staff ensure that right equipment is available and that participants are rightly prepared for the task: participants have a warm sweater, and they are not hungry or suffering from sleep deprivations
Participant starts feeling cold	Take a break during the hike/kayaking to adjust cloths
Participant shows symptoms of being cold, shivering, and blue lips	Take actions to warm the participant. Change to dry warm cloths, give hot beverages
Hypothermia	Call for medical treatment 112, prepare evacuation.

9.2 Risk exposure/consequence matrix of the WAYA study

Risk Categories	Description of risk	Prevention and measures	Score: Likelihood – consequence*
**Participant-related:**			
**COVID-19 infection**	Risk for COVID-19 infection travelling to and during intervention	Following FHM and Governmental regulations:<br>- not travel/participate when they have symptoms<br>- social distancing<br>- face masks where it is not possible to keep safe distance<br>- hygiene, disinfection	**Score: 6-12**<br><br>Possible<br><br>Mild-Severe
	Risk for COVID-19 infection travelling to and during the intervention	Identification of infections and preventive measures:<br>- vaccination<br>- face mask during car/bus/train/flight transport<br>- COVID-19 antigen testing of participants and facilitators<br>- own tent in wilderness program<br>- own hotel room in holiday program<br>- separate hotel dining room	**Score: 6-12**<br><br>Possible<br><br>Mild-Severe
	Symptoms of COVID-19 during the intervention	With occurrence of symptoms, direct antigen testing. Depending on the outcome:<br>- a PCR test at test site of the Region Västernorrland<br>- testing of others in the group<br>- isolation in hotel room (holiday program)<br>- evacuation to guesthouse for stay during the isolation period (wilderness program)<br>- with severe symptoms, contact primary/secondary healthcare<br>- consultation with medical adviser of the WAYA study were needed	**Score: 4-8**<br><br>Less likely<br><br>Mild-Severe
**Personal data breach**	Risk that personal (sensitive) data of participants in the study becomes publicly available.	Personal data of participants will only be known to the PI and CPI in the study and handled in according to the Clinical Trials Regulation (EU Regulation No. 536/2014) and the General data protection Regulation (EU regulation No. 2016/679). Facilitators will keep all information that is shared during the intervention confidential. Dissemination of any kind of information on the study can	**Score: 4**<br>Unlikely<br><br>Severe

		only be done after permission of the PI and/or respective participant.	
**Poor physical condition**<br>- related to not being trained / experienced to be outdoors	Blister/sore	Information on physical condition of participants, safety instructions, sufficient breaks, disinfection, bandage, tape, assessment and monitoring how it affects their ability to continue participation	**Score: 6**<br>Possible<br><br>Mild
	Wound/cut	Information on physical condition of participants, safety instructions, sufficient breaks, hiking with Nordic walking sticks, head lights, bandage, tape, assessment and monitoring how it affects their ability to continue participation	**Score: 6-9**<br>Possible<br><br>Mild-Noticeable
	Sprain	Information on physical conditions of participants, safety instructions, sufficient breaks, support bandages to prevent or treat sprains, head lights, hiking with Nordic walking sticks, assessment and monitoring how it affects their ability to continue participation	**Score: 6-9**<br>Possible<br><br>Mild-Noticeable
	Fracture, large wound, concussion	Information on physical condition of participants, safety instructions, sufficient breaks, head lights, hiking with Nordic walking sticks, assessment of the situation, first aid, (emergency) evacuation by foot, carrier, car, ambulance, helicopter	**Score: 6-8**<br>Less likely<br><br>Noticeable-Severe
**Poor physical condition**<br>- related to medical condition / treatment	Nausea, headache, stomachache, cold, sore throat, diarrhea, constipation, allergic reaction, dizziness, insomnia, feeling cold, physical exhaustion	Information on physical condition of participants, safety instructions, sufficient breaks, balance in program activities and leisure time, hiking with Nordic walking sticks, walking/moving before going to sleep, warm water bottle, use of medication (paratec, ibuprofen, antihistamines, anti-diarrheal medication, laxatives, nasal spray, lozenges), assessment and monitoring how it affects their ability to continue participation	**Score: 6-9**<br>Possible<br><br>Mild-Noticeable
	Anaphylactic shock	Safety instructions, information on known allergies, protection measures against known allergies, epinephrine injection, assessment of the situation, (emergency) evacuation by foot, carrier, car, ambulance, helicopter	**Score: 8**<br>Less likely<br><br>Severe
**Poor mental health**<br>- related to medical	Anxiety/fear, exhaustion,	Information on mental condition of participants, safety instructions, sufficient breaks, balance in program	**Score: 6-9**<br>Possible

condition / treatment	depression, brain fatigue	activities and leisure time, coaching, assessment and monitoring how it affects their ability to continue participation	Mild-Noticeable
**Intoxication**	Alcohol, drugs, medication or other	Information on possible addictions of participants, instructions on non-alcohol/drugs policy during the interventions, assessment and monitoring how it affects their ability to continue participation or evacuation where needed	**Score: 4-8** Less likely<br><br>Mild-Severe
**Missing participant(s)**	Participant(s) getting lost in nature or not returning to the hotel	Safety instructions, facilitator at the end of the group when hiking, facilitator at the front and end of the group when kayaking, frequent group counting, pairing-up participants (keep an eye on- and inform your Trangia buddy or Spa buddy), mapping and compassing, telephone contact, initiate searches	**Score: 4-8** Less likely<br><br>Mild-Severe
**Loss of medication**	Necessary medication for participants is lost, or cannot be kept cold due to unforeseen circumstances	Discuss and document each participants' s necessary medication, assure conditions for safe transport, and have an individual action plan to provide for the necessary medication via the nearest pharmacy	**Score: 8** Less likely<br><br>Severe
**Environment-related:**			
**Frequent insect/animal exposure**	Insect or snake bite, wasp sting, tick	Safety instructions, daily tick control, insect repellent, mosquito hat net, anti-sting, disinfection, tick-removal tool, assessment and monitoring how it affects their ability to continue participation, first aid, (emergency) evacuation by foot, carrier, car, ambulance, helicopter	**Score: 8-12** Possible<br><br>Mild-Severe
**Fallen trees, branches sticking out**	Wound/cut	Safety instructions, hiking with Nordic walking sticks, head lights, bandage, tape, assessment and monitoring how it affects their ability to continue participation	**Score: 6-9** Possible<br><br>Mild-Noticeable
**Contaminated water, poisonous berries, mushrooms or other**	Food/water poisoning	Safety instructions on water treatment (filtering/cooking) and what can be eaten and cannot be eaten in nature, and to check with others before eating something from nature	**Score: 8** Less likely<br><br>Severe
**Sunny weather conditions**	Sunburn	Safety instructions, use of sunscreen and hat/cap, assessment and monitoring how it affects their ability to continue participation	**Score: 6** Possible<br><br>Mild

**Cold and rainy weather conditions**	Sprain	Safety instructions, decision for alternative hiking tracks, support bandages to prevent or treat sprains, head lights, hiking with Nordic walking sticks, assessment and monitoring how it affects their ability to continue participation	**Score: 6-9**<br>Possible<br><br>Mild-Noticeable
	Hypothermia	Safety instructions, rain clothes, warm clothes, tarp to protect from the rain, stove in the tentipi, hot blanket	**Score: 8**<br>Less likely<br><br>Severe
**Falling/slipping during gorge climbing**	Risk of slipping/falling during climbing, prusik knot slips on wet safety rope, risk of falling and hitting tree or another participant	Participants will use a short cord connected to the safety rope to prevent falling for more than a few centimeters; instructions for how to use the safety rope, the short cord should be attached with a prusik knot and should be checked for slipping, as well that it should always be above the waist while climbing. Only 2 participants per rope, the climb will be divided in two sections with both one safety rope. One instructor will be attached to an additional safety rope, positioned in the middle to help participants in case they experience difficulties.	**Score: 4-6**<br>Less likely<br><br>Mild-Noticeable
**Strong wind currents – waves exposure upon kayaking**	Hypothermia	With expected strong wind and waves, there will be no sea-kayaking, alternative transport to the Island by boat, other safety instructions include dry or wetsuits and heat blanket.	**Score: 8**<br>Less likely<br><br>Severe
	Drowning	With expected strong wind and waves, there will be no sea-kayaking, alternative transport to the Island by boat, other safety instructions include life-vest and whistle	**Score: 8**<br>Less likely<br><br>Severe
**Equipment-related:**			
**Poor hiking shoes, outdoor clothes**	Blister/sore	Ensuring and checking good quality equipment, breaking in of hiking shoes, disinfection, bandage, tape, assessment and monitoring how it affects their ability to continue participation	**Score: 4**<br>Less likely<br><br>Mild
	Sprain	Ensuring and checking good quality equipment, breaking in of hiking shoes, support bandages to prevent or treat sprains, head lights, hiking with Nordic walking sticks, assessment and	**Score: 4-6**<br>Less likely<br><br>Mild-Noticeable

		monitoring how it affects their ability to continue participation	
**Poor sea kayaking equipment**	Hypothermia or Drowning	Ensuring and checking good quality of kayaks, life vests, dry or wet suits.	**Score: 4**<br>Unlikely<br><br>Noticeable-Severe
**Inexperience with outdoor equipment**	Burn, due to accident with boiling water, burnable liquid from camping stove or from camping fire	Safety instructions, equipment instructions, cooling with water, bandage, assessment and monitoring how it affects their ability to continue participation	**Score: 6-9**<br>Possible<br><br>Mild-Noticeable
	Wound/cut, due to inexperience with outdoor equipment	Safety instructions, equipment instructions, head lights, bandage, tape, assessment and monitoring how it affects their ability to continue participation	**Score: 6-9**<br>Possible<br><br>Mild-Noticeable

*Risk scores: 1-4 (green) acceptable risk; 6-8 (yellow) acceptable risk but requires close monitoring; 9-12 (orange) acceptable risk, but requires close monitoring to prevent the risk, follow-up, and evacuation where necessary; 16 (red) not acceptable risk, should be prevented at any costs and in case it happens it requires immediate evacuation.

10 Equipment list related to the risks identified

**Item for wilderness program**	**Number**	**Personal safety equipment**	**Shared safety equipment**	**Responsible equipment manager**
Compass	25	X		
Map	32	X		
Water filter	2		X	
Cooking gear/fuel	20		X	
Matches	50	X		
Sunscreen	5		X	
Tentipi / stove	1		X	
Tarps	1		X	
Ropes	3		X	
Climbing belts	10		X	
Prescription medication (individual participants)	20	X		
Pain and (other)medication: antihistamine, anti-diarrheal medication, laxative, nasal spray, lozenge	Variable: more packages of pain medication than other medication		X	
Rescue telephone	2		X	
COVID-19 test cassettes	200		X	

Wet suit / dry suit / life vest	17		X	
Heat blanket	2		X	
Warm clothes	25	X		
First aid kit	30	X		
Whistle	17	X		
Face mask	200	X		
Water bottles	32	X		
Disinfection gel	15		X	
Disinfection wipes	80	X		
Tick removal tool	4		X	
Nordic hiking sticks	10	X		
Insect repellent	10		X	
Anti-bite	10		X	
Mosquito hat net	10	X		
Headtorch (batteries)	17	X		
Epinephrine injection	3		X	

11 Safety Instructions participants

Mid Sweden University will provide all necessary outdoor equipment, outdoor clothing, and footwear for each participant. All equipment needed to prevent risks, and measures to deal with injuries and ailments will be provided by Mid Sweden University as well.

Participants will receive the following safety instructions in the wilderness program:
- what to bring (appropriate other clothing, personal medication, other)
- actions/measures to prevent that they get lost
- actions/measures what to do if they are lost
- daily self-check for ticks
- proper use of outdoor equipment
- mapping/compassing, navigation
- weather condition (wind, sun, rain, cold, lightening)
- basics of first aid
- terrain conditions: alertness for slippery areas, fallen trees, branches, lose stones
- making fire and cooking
- cooking/filtering drinking water and drinking enough
- pooping/peeing
- morning stretching
- how to stay warm in the night
- importance of frequent rests and breaks
- take turns in leading of the group and sharing decision-making responsibilities
- kayaking (getting in and out of the kayak, paddling, stay together, close to shore etc)
- match-up with a body
- making knots
- protection against insects, bugs, snakes
- food, eating in time and enough
- disposal of trash
- what to eat and what not to eat in nature
- practice good hygiene

12 Safety monitoring

The facilitators of all four intervention programs in the summer and 3-months follow-up program will closely monitor whether any adverse events/effects (accidents/incidents, medical issue/problems) have occurred. Adverse events will be documented in the preprinted field note diaries of facilitators and discussed at the daily debriefing meeting during the program. In addition to adverse events, facilitators will also monitor close calls/near misses and report them in their field note diary for discussion during the daily debriefing meeting.

After the intervention, the PI will enter the adverse events in the Case Report Forms (CRF) of the respective participants and possible causality with the study intervention will be assessed based on the information in the field note diaries and evaluations of debriefing meetings. Adverse events will be coded according to the Medical Dictionary for Regulatory Activities (MedDRA, 2007) by the CPI, and analyzed using preferred terms and allocation to system organ class. Safety of the interventions will be measured by analyzing number and type of adverse events reported.

13 References

Clinical Trials Regulation (EU Regulation No. 536/2014). European Medicines Agency. Accessed on 29 April 2021: https://www.ema.europa.eu/en/human-regulatory/research-development/clinical-trials/clinical-trial-regulation

General data protection Regulation (EU regulation No. 2016/679). Accessed on 29 April 2021: https://eur-lex.europa.eu/eli/reg/2016/679/oj

Jong M, Lown EA, Schats W, Mills ML, Otto HR, Gabrielsen LE, Jong MC. (2021) A scoping review to map the concept, content, and outcome of wilderness programs for childhood cancer survivors. PLoS ONE 16(1): e0243908. https://doi.org/10.1371/journal.pone.0243908

Medical Dictionary for Regulatory Activities (MedDRA®) (2007). Pharmacovigilance, ed. E.A. Mann RD.

Svenska Fjällklubben, Säkerhetsplan, version 1.3. Accessed on 29 April 2021: https://fjallklubben.se/images/SFK/Sakerhetsplanen/SFK_Sakerhetsplan_1.3_Bilaga_2_Riskanalys.pdf2021

14 Appendix I: Emergency and Evacuation procedures

Wilderness program

Table 1. Emergency and evacuation procedure in case of an accident/incident
Day 1/2: Group 1: 14/15 June and Group 2: 27/28 June 2021
Glosand (Härnösand): Coordinates N 6933196, E 646543 (SWEREF 99 TM)

**Action**	**Responsible facilitator**	**Action**	**Responsible facilitator**
**Affected participant (s)**		**Rest of the group of participants**	
1. Discuss/assess the situation		1. Taking charge of the group	
2. In case of a missing person, initiate and coordinate searches		2. Decide whom of the participants/facilitators can support in searches	
3. Approach and stay with the (found) participant on site		3. Decide whether to continue as a group, wait or set-up camp	
4. First aid: assess and stabilize condition participant		4. Ensure all group members are OK	
5. Note down necessary information of the accident/incident and condition of the participant in the field diary		5. Maintain group morale as much as possible	
6. Discuss/assess need for evacuation		6. Coach/guide in case of emotions, anxiety among other participants	
7. Contact / discuss with medical advisor WAYA study if needed			
8. Assess the type of evacuation:<br>- 5 minutes' walk/transport to car<br>- 25 minutes' drive to primary care in Härnösand<br>- 35 minutes' drive to hospital in Sundvall			
9. Emergency call if needed for evacuation			
10. Accompany participant upon evacuation			

Table 2. Emergency and evacuation procedure in case of an accident/incident
Day 2/3: Group 1: 15/16 June and Group 2: 28/29 June 2021
Dalsjön (Käxed, Kramfors): Coordinates N 7000417, E 672355 (SWEREF 99 TM)

**Action**	**Responsible facilitator**	**Action**	**Responsible facilitator**
**Affected participant (s)**		**Rest of the group of participants**	
1. Discuss/assess the situation		1. Taking charge of the group	
2. In case of a missing person, initiate and coordinate searches		2. Decide whom of the participants/facilitators can support in searches	
3. Approach and stay with the (found) participant on site		3. Decide whether to continue as a group, wait or set-up camp	
4. First aid: assess and stabilize condition participant		4. Ensure all group members are OK	
5. Note down necessary information of the accident/incident and condition of the participant in the field diary		5. Maintain group morale as much as possible	
6. Discuss/assess need for evacuation		6. Coach/guide in case of emotions, anxiety among other participants	
7. Contact / discuss with medical advisor WAYA study if needed			
8. Assess the type of evacuation:<br>- 15-60 minutes' walk/transport to road with a car (depending on hiking location to and from Dalsjön)<br>- From the road 25 minutes' drive to primary care in Örnsköldsvik<br>- From the road 25 minutes' drive to hospital Örnsköldsvik			
9. Emergency call if needed for evacuation			
10. Accompany participant upon evacuation			

Table 3. Emergency and evacuation procedure in case of an accident/incident
Day 3-5: Group 1: 16-18 June and Group 2: 29 June – 1 July 2021
Kälaviken (Käl, Kramfors): Coordinates N 6999825, E 677273 (SWEREF 99 TM)

**Action**	**Responsible facilitator**	**Action**	**Responsible facilitator**
**Affected participant (s)**		**Rest of the group of participants**	
1. Discuss/assess the situation		1. Taking charge of the group	
2. In case of a missing person, initiate and coordinate searches		2. Decide whom of the participants/facilitators can support in searches	
3. Approach and stay with the (found) participant on site		3. Decide whether to continue as a group, wait or set-up camp	
4. First aid: assess and stabilize condition participant		4. Ensure all group members are OK	
5. Note down necessary information of the accident/incident and condition of the participant in the field diary		5. Maintain group morale as much as possible	
6. Discuss/assess need for evacuation		6. Coach/guide in case of emotions, anxiety among other participants	
7. Contact / discuss with medical advisor WAYA study if needed			
8. Assess the type of evacuation:<br>- 15-60 minutes' walk/transport to road (depending on hiking location to Kälaviken)<br>- From the road 25-30 minutes' drive to primary care in Örnsköldsvik<br>- From the road 25-30 minutes' drive to hospital in Örnsköldsvik			
9. Emergency call if needed for evacuation			
10. Accompany participant upon evacuation			

Table 4. Emergency and evacuation procedure in case of an accident/incident
Day 5-7: Group 1: 18-20 June and Group 2: 1-3 July 2021
Baggsandsbukten (Mjältön, Kramfors): Coordinates N 6994059, E 677999 (SWEREF 99 TM)

**Action**	**Responsible facilitator**	**Action**	**Responsible facilitator**
**Affected participant (s)**		**Rest of the group of participants**	
1. Discuss/assess the situation		1. Taking charge of the group	
2. In case of a missing person, initiate and coordinate searches		2. Decide whom of the participants/facilitators can support in searches	
3. Approach and stay with the (found) participant on site		3. Decide whether to continue as a group, wait or set-up camp	
4. First aid: assess and stabilize condition participant		4. Ensure all group members are OK	
5. Note down necessary information of the accident/incident and condition of the participant in the field diary		5. Maintain group morale as much as possible	
6. Discuss/assess need for evacuation		6. Coach/guide in case of emotions, anxiety among other participants	
7. Contact / discuss with medical advisor WAYA study if needed			
8. Assess the type of evacuation:<br>- 1-60 minutes' walk/transport to boat (depending on the hiking trips on Mjältön)<br>- 10 minutes' boat transport to road at Skulebergets Havscamping<br>- From the road 20 minutes' drive to primary care in Örnsköldsvik<br>- From the road 20 minutes' drive to hospital in Örnsköldsvik			
9. Emergency call if needed for evacuation			
10. Accompany participant upon evacuation			

Table 5. Emergency and evacuation procedure in case of an accident/incident
Day 7-8: Group 1: 20-21 June and Group 2: 3-4 July 2021
Sund (Kramfors): Coordinates N 6995006, E 674620 (SWEREF 99 TM)

**Action**	**Responsible facilitator**	**Action**	**Responsible facilitator**
**Affected participant (s)**		**Rest of the group of participants**	
1. Discuss/assess the situation		1. Taking charge of the group	
2. In case of a missing person, initiate and coordinate searches		2. Decide whom of the participants/facilitators can support in searches	
3. Approach and stay with the (found) participant on site		3. Decide whether to continue as a group, wait or set-up camp	
4. First aid: assess and stabilize condition participant		4. Ensure all group members are OK	
5. Note down necessary information of the accident/incident and condition of the participant in the field diary		5. Maintain group morale as much as possible	
6. Discuss/assess need for evacuation		6. Coach/guide in case of emotions, anxiety among other participants	
7. Contact / discuss with medical advisor WAYA study if needed			
8. Assess the type of evacuation:<br>- 1 minutes' walk/transport to road<br>- From the road 30 minutes' drive to primary care in Örnsköldsvik<br>- From the road 30 minutes' drive to hospital in Örnsköldsvik			
9. Emergency call if needed for evacuation			
10. Accompany participant upon evacuation			

Holiday program

Table 6. **Emergency and evacuation procedure in case of an accident/incident**
Day 1-8: Group 3: 5-12 July and Group 4: 15-22 July 2021
Hotell Södra Berget, Södra Stadsberget 1, 852 38 Sundsvall (tel: +46 60 67 10 00)

**Action**	**Responsible facilitator**	**Action**	**Responsible facilitator**
**Affected participant (s)**		**Rest of the group of participants**	
1. Discuss/assess the situation		1. Taking charge of the group	
2. In case of a missing person, initiate and coordinate searches		2. Decide whom of the participants can support in searches	
3. First aid: assess and stabilize condition participant		3. Ensure all group members are OK	
4. Note down necessary information of the accident/incident and condition of the participant in the field diary		4. Maintain group morale as much as possible	
5. Discuss/assess need for evacuation		5. Coach/guide in case of emotions, anxiety among other participants	
6. Contact / discuss with medical advisor WAYA study if needed			
7. Assess the type of evacuation			
8. Emergency call if needed for evacuation			
9. Accompany participant upon evacuation to primary care or hospital in Sundsvall			
