# Supplementary material for: The Development and Acceptability of a Wilderness Programme to Support the Health and Well-Being of Adolescent and Young Adult Cancer Survivors: The WAYA Programme
Source: Int J Environ Res Public Health. 2022 Sep 22;19(19):12012. doi: 10.3390/ijerph191912012 (PMC9566286; doi:10.3390/ijerph191912012)
Supplement: Supplementary file 1 [file ijerph-19-12012-s001.zip › supplementary/Supplementary file S3 Focus group.pdf]

Focus group: Facilitator Evaluation WAYA Wilderness Program

29 November 2021

1. **Type of Wilderness Program**

Expedition (8-days under the summer) versus base-camp (4-days in the fall)

- What did you experience were advantages/disadvantages of the WAYA expedition or/and the WAYA base-camp?

2. **Activities in the Program**

Groups activities versus individual-adapted activities

- What is your opinion on the fact that the WAYA study offered individual-adapted activities in addition to activities with the whole group, dependent on participants capability/wishes? Or similar to this: Does diversity of the group (related to physical/mental capabilities of participants) seem to be more important than homogeneity, or the other way around?

Participation in program activities versus free (own) time

- What are your reflections on the balance in the WAYA program for participants related to program-activities versus free (own) time?

Activities under supervision by facilitators versus activities on their own.

- How much time in the WAYA program should allow participants to do activities on their own, without supervision by any facilitator?

Type of activities

- How did you experience the variance in activities in the WAYA program: hiking, backpacking, camping, sea-kayaking, rock climbing, mindfulness exercises, nature experience, bush-craft skills (what should be left out or offered less, what else should be included, or should be offered more?)

3. **Safety of the Program**

- How did you experience the overall safety of the WAYA program for participants?

4. **Facilitators of the Program**

- How did you experience the facilitator – participant ratio in the WAYA program?

- What is your opinion about the qualifications/competences and leadership of the facilitators in the WAYA program?

- What is your opinion on facilitators being part /sharing their experiences in the mindfulness exercises and reflections?

- How do you reflect upon the clarity of your task/role as facilitator in the program?

- How did you experience the communication between the facilitators and what could be improved?

5. Equipment in the Program

- What is your opinion on the outdoor equipment that was provided to the participants in the program?

- What is your opinion on the food that was provided to the participants in the program?
