# Supplementary material for: The Development and Acceptability of a Wilderness Programme to Support the Health and Well-Being of Adolescent and Young Adult Cancer Survivors: The WAYA Programme
Source: Int J Environ Res Public Health. 2022 Sep 22;19(19):12012. doi: 10.3390/ijerph191912012 (PMC9566286; doi:10.3390/ijerph191912012)
Supplement: Supplementary file 1 [file ijerph-19-12012-s001.zip › supplementary/Supplementary file S4 Interview guide.pdf]

WAYA study: Interview guide

The interviews will be conducted with an open thematic structure where participants first will be asked to briefly describe / tell about their experiences in connection with participation in the WAYA program and how they look at it now, after more than three months after the expedition, and a few weeks after base-camp. Furthermore, they are asked to reflect in depth on the context of the program, the other participants, activities, and facilitators.

Conducting interviews from a thematic perspective provides an opportunity for follow-up questions such as example: what do you mean by that, please describe it more, how did you think, what did you experience, what do you think what made you feel that way, etc.

The thematic approach gives a direction linked to what we have as the purpose of the interviews, however depending on what the participants bring up, other issues can be opened up and touched upon.

- <u>General impression of participating in the program:</u>

o Please describe your general feeling/experience of being a part of and participating in the program…

o Please describe what was the best of the program…

o Please describe what was not so good, or what you think can be improved…

- <u>You experience of the program activities</u>

o Hiking

- Easy/difficult/strenous

o Camping

- How did you sleep?
- How did you experience to set-up/break down camp?
- To cook?
- Equipment such as clothing, shoes and dirt, hygiene, dry, cold, wetness?

o Please describe your experiences with the different activities

- Easy, difficult, challenging, scary, impossible……

o Please describe what made you choose to participate in the different activities?

o Please describe what made you choose not to participate (if you did)

o Was there something that was too difficult?

o What kind of experiences / feelings arose in connection with the activities?

o What was the most fun / satisfying activity?

o Please describe the least fun, negative or difficult activity?

- <u>The facilitators</u>

o Please describe how you perceived the different facilitators in the program

o In what way did you feel that they supported you while participating in the program?

o Can you give some tips on how they can be more supportive?

- <u>The other participants</u>

o Please describe in general how you feel about the other participants?

- Could you make contact / friendship
- Difficult / easy
- In what way did you feel connected to them? (or not)
- In what way did they respect you?
- Did they listen to what you had to say?
- Were you taken seriously?
- Did you listen to the others?
- Did you take others seriously?
- Positive experiences of social interaction?
- Negative experiences of social interaction?

- <u>NATURE - High Coast area</u>

o Please describe a little about your general experiences of being out in nature?

o What is your feeling of the area where the program was implemented?

o Were there any specific places you felt more comfortable than others?

o Was there any time during the hike / camping that you felt scared or anxious

for nature?

- How did you handle that?

- <u>Health aspects</u>

o Is there anything in relation to your health and well-being that you feel has changed during or after the nature stay?

- <u>Risk/Accidents/Injuries</u>

o Was there any time under program participation that you did not feel safe?

- If so, when? Why and how did you get through it?

- Did you get any injuries during the program?
- If so, what happened, and how did you deal with it?

o Was there anything that happened that you did not tell/inform the facilitators about?

- If so, what happened, and why did you choose not to inform them?

- <u>Motives for participation</u>

o Why did you choose to participate in this program?

o If you had to choose a group,

- Would you rather have been in the other group? If so, why?

o Which expectations did you have of the program?

o Was it what you expected? If not, why?

- <u>Would you recommend other friends to sign up to participate in a program like this?</u>

o If so, why?
